# Machine learning modeling of genome-wide copy number alteration signatures reliably predicts IDH mutational status in adult diffuse glioma

**DOI:** 10.1186/s40478-021-01295-3

**Published:** 2021-12-04

**Authors:** Nicholas Nuechterlein, Linda G. Shapiro, Eric C. Holland, Patrick J. Cimino

**Affiliations:** 1 Paul G. Allen School of Computer Science & Engineering, University of Washington, Seattle, WA USA; 2 Division of Human Biology, Fred Hutchinson Cancer Research Center, Seattle, WA USA; 3 Department of Laboratory Medicine and Pathology, Division of Neuropathology, University of Washington, 325 9th Avenue, Box 359791, Seattle, WA 98104 USA

**Keywords:** Adult diffuse glioma, Glioblastoma, Astrocytoma, Oligodendroglioma, Copy number, IDH, Isocitrate dehydrogenase, TCGA, REMBRANDT, GLASS

## Abstract

Knowledge of 1p/19q-codeletion and *IDH1/2* mutational status is necessary to interpret any investigational study of diffuse gliomas in the modern era. While DNA sequencing is the gold standard for determining IDH mutational status, genome-wide methylation arrays and gene expression profiling have been used for surrogate mutational determination. Previous studies by our group suggest that 1p/19q-codeletion and IDH mutational status can be predicted by genome-wide somatic copy number alteration (SCNA) data alone, however a rigorous model to accomplish this task has yet to be established. In this study, we used SCNA data from 786 adult diffuse gliomas in The Cancer Genome Atlas (TCGA) to develop a two-stage classification system that identifies 1p/19q-codeleted oligodendrogliomas and predicts the IDH mutational status of astrocytic tumors using a machine-learning model. Cross-validated results on TCGA SCNA data showed near perfect classification results. Furthermore, our astrocytic IDH mutation model validated well on four additional datasets (AUC = 0.97, AUC = 0.99, AUC = 0.95, AUC = 0.96) as did our 1p/19q-codeleted oligodendroglioma screen on the two datasets that contained oligodendrogliomas (MCC = 0.97, MCC = 0.97). We then retrained our system using data from these validation sets and applied our system to a cohort of REMBRANDT study subjects for whom SCNA data, but not IDH mutational status, is available. Overall, using genome-wide SCNAs, we successfully developed a system to robustly predict 1p/19q-codeletion and IDH mutational status in diffuse gliomas. This system can assign molecular subtype labels to tumor samples of retrospective diffuse glioma cohorts that lack 1p/19q-codeletion and IDH mutational status, such as the REMBRANDT study, recasting these datasets as validation cohorts for diffuse glioma research.

**Supplementary Information:**

The online version contains supplementary material available at 10.1186/s40478-021-01295-3.

## Introduction

Diffuse gliomas comprise the most common adult malignant tumors of the central nervous system [49]. These adult diffuse gliomas consist of three major biologically and clinically distinct molecular subtypes, which are defined by the mutational status of *isocitrate dehydrogenase 1* and *2* (IDH) and the presence or absence of co-deletion of whole chromosome arms 1p and 19q, which further stratifies IDH-mutant diffuse glioma [39, 40]. These genetic alterations are strong predictors of survival and contain more information than historical histologically-based classification and grading systems [9, 15, 38, 52, 58]. In routine surgical neuropathology, it is common practice to classify diffuse gliomas in terms of IDH mutational and 1p/19q-codeletion status. Likewise, in contemporary research studies of adult diffuse gliomas there is limited utility, if any, in gaining insights into the biology of gliomas if the samples are not well annotated for this molecular information. Furthermore, determining the robustness and reliability of any findings in human gliomas requires testing and validation across multiple cohorts. Consequently, older retrospective cohorts of diffuse gliomas lacking IDH mutational and 1p/19q-codeletion status have limited utility for validating contemporary adult diffuse glioma study results.

As a testing platform, DNA sequencing is the gold standard method to detect the spectrum of clinically relevant canonical and non-canonical IDH mutations [11, 21, 55, 57, 59]. More recently, methods have been developed to infer IDH mutation and 1p/19q-codeletion status from methylation array [10, 47], gene expression [12], and magnetic resonance imaging data [3, 35, 37, 43], which can provide surrogate molecular subtype labels for validating adult diffuse glioma study results. We have previously observed that there is a strong association between adult diffuse glioma molecular subtype and patient somatic copy number alteration (SCNA) profiles, indicating that SCNA data alone may reflect global genomic structures that are associated with, and predictive of, IDH mutational status [7, 16, 17, 48]. Furthermore, SCNA data has the advantage of directly encoding the extent of 1p and 19q loss, although an empirical threshold necessary to definitively call 1p/19q-codeletions has yet to be established.

In this study, we sought to develop and evaluate a robust system that predicts adult diffuse glioma IDH mutational status and 1p/19q-codeletion status from SCNA data alone. Special care is given to establish appropriate thresholds for calling 1p/19q-codeletions as well as simultaneous gain of whole chromosome 7 and loss of whole chromosome 10 (+ 7/ − 10), the latter of which is necessary for molecular grading of IDH-wildtype diffuse astrocytic gliomas [8]. We validate our system on a TCGA holdout set of histological World Health Organization (WHO) grade 4 tumors and three additional independent diffuse glioma datasets, including a dataset published by Glioma Longitudinal AnalySiS Consortium (GLASS) [6, 10, 18, 31]. Finally, we report the system’s predictions on the retrospective REMBRANDT study, where genome-wide SCNA data is available, but IDH sequencing is not [28]. Overall, our study makes older adult diffuse glioma datasets with SCNA data but without molecularly diagnoses better suited for validating contemporary findings. Additionally, this study proposes evidence-based thresholds for 1p/19q-codeletions and + 7/ − 10.

## Materials and methods

### TCGA glioma dataset

Somatic mutation calls for The Cancer Genome Atlas (TCGA) glioblastomas and lower-grade astrocytic and oligodendroglial tumors (N = 812) computed by the Multi-Center Mutation Calling in Multiple Cancers (MC3) project [20] were downloaded from University of California Santa Cruz (UCSC) Xena (https://xena.ucsc.edu/) [26]. Three versions of TCGA gene-level glioma somatic copy number alteration (SCNA) calls were either downloaded from UCSC Xena or computed from copy number segmentation files downloaded from the National Cancer Institute’s Genomic Data Commons (GDC) Data Portal (https://gdc.cancer.gov/). TCGA SCNA data downloaded from UCSC Xena (UCSC hg19) was the thresholded output of the Genomic Identification of Significant Targets in Cancer 2.0 (GISTIC) algorithm aligned to human genome assembly GRCh37 (hg19) [46]. The GISTIC algorithm assigns each gene a discrete value between − 2 and 2 corresponding to gene homozygous deletion (− 2), single copy deletion (− 1), diploid normal copy (0), low-level amplification (1), or high-level amplification (2). We apply GISTIC to the copy number segmentation files downloaded from the GDC Data Portal to generate two additional versions of TCGA glioma SCNA data. One version (GDC hg19) was aligned to hg19 and the other (GDC hg38) to human genome assembly GRCh38 (hg38). TCGA SCNA data is derived from Affymetrix SNP 6.0 arrays.

### TCGA glioma ultra-low-pass whole genome sequencing dataset

TCGA data was processed as ultra-low-pass whole genome sequencing (ULP-WGS, 0.1x) to compare Affymetrix SNP 6.0 array derived SCNA data to lower coverage data. Bam files for every patient in the TCGA-LGG and TCGA-GBM projects with available bam files were downloaded from the GDC. These bam files were realigned to hg19 using SAMtools [36], BEDTools [51], Bowtie 2 [34], the Picard Toolkit (version 2.7.1) [56], and the Genome Analysis Toolkit (GATK, version 3.7) [45]. The R package *HMMcopy* (version 1.36.0) [33] was used to create wig files, and the R package *ichorCNA* [1] was used to create a custom panel of normals from bam files of normal blood or tissue downloaded from GDC for patients in the TCGA-LGG and TCGA-GBM projects. *ichorCNA* was then used to create copy number segmentation files using wig files created by *HMMcopy*. GISTIC was used to compute gene-level SCNA calls from these segmentation files.

### TCGA molecular subtype training labels

Our baseline IDH mutation and 1p/19q-codeletion training labels are derived from IDH mutation calls reported by the MC3 project [20] and 1p/19q-codeletion annotations published by Ceccarelli et al. [12]. We compared the MC3 IDH mutation labels to the IDH mutation labels published by Ceccarelli et al. and the 1p/19q-codeletion status published by Ceccarelli et al. to the gene-level TCGA SCNA training data (Additional file 1: Table 1). Two patients (TCGA-06-0151, TCGA-HT-A618) called IDH-wildtype in the MC3 project are labeled as IDH-mutant astrocytomas by Ceccarelli et al. One patient (TCGA-06-0151) is histological WHO grade 4, harbors + 7/ − 10, and lacks mutations in *TP53* and *ATRX*. Because these are all characteristics of IDH-wildtype glioblastoma, we maintain the MC3 IDH-wildtype designation. The other patient (TCGA-HT-A618), however, is histological WHO grade 3, has intact chromosomes 7 and 10, and harbors *TP53* and *ATRX* mutations; thus, we replace the MC3 project IDH-wildtype label with the Ceccarelli et al. IDH-mutant astrocytoma designation. Conversely, one patient (TCGA-P5-A72U) that carries an IDH mutation in the MC3 project data is labeled IDH-wildtype by Ceccarelli et al. This patient harbors + 7/ − 10, and lacks mutations in *TP53* and *ATRX*, and we therefore consider this an IDH-wildtype glioblastoma. While SCNA data from three TCGA SCNA pipelines indicate all 171 Ceccarelli et al. oligodendroglial designated tumors carry 1p/19q-codeletions, five Ceccarelli et al. designated IDH-mutant astrocytomas carry significant loss of 1p and 19q without loss of either 1q or 19p on at least one, but not all, of the three TCGA SCNA versions we used (UCSC hg19, GDC hg19, GDC hg38) (Additional file 2: Fig. 1). Furthermore, all five tumors are *TP53*-wildtype, *ATRX*-wildtype, and are classified histopathologically as oligodendroglioma or mixed oligoastrocytoma, indicating that they may be oligodendrogliomas (Additional file 1: Table 2). However, to avoid uncertainty, we excluded these five tumors from this study.

### TCGA training set

Our final TCGA training set consists of 786 adult diffuse gliomas, 171 of which are IDH-mutant and 1p/19q-codeleted oligodendrogliomas, 257 of which are IDH-mutant astrocytomas, and 358 of which are IDH-wildtype glioblastomas (Additional file 1: Table 3). Apart from the five Ceccarelli et al. designated IDH-mutant tumors we justified excluding above, we also excluded 21 histological lower-grade IDH-wildtype tumors, because we could not confirm that they harbored simultaneous gain of whole chromosome 7 and loss of whole chromosome 10 (+ 7/ − 10), *EGFR* amplification, or TERT promoter (*TERT*p) mutation required by the fifth edition of the WHO criteria for classification as adult diffuse gliomas [8, 40]. The exclusion of these tumors is further justified in the main text. The remaining 70 histological lower-grade IDH-wildtype tumors showed molecular markers consistent with the updated definition of IDH-wildtype glioblastoma as described in the fifth edition of the WHO criteria for classification as adult diffuse gliomas.

### TCGA validation set

In addition to the 786 adult diffuse gliomas in our TCGA training set and the 26 tumors we excluded from our study, 167 other TCGA tumors have IDH mutational and 1p/19q-codeletion status annotations published by Ceccarelli et al., although they do not have MC3 mutational data to confirm these annotations. This cohort of patients consists of 2 oligodendrogliomas, 17 histological grade 4 IDH-mutant astrocytomas, 146 histological grade 4 IDH-wildtype glioblastomas, and 2 histological lower-grade IDH-wildtype tumors that do not qualify as adult diffuse IDH-wildtype glioma under the criteria in the fifth edition of the WHO classification of CNS tumors [40, 41]. We restrict this cohort to the 163 grade 4 astrocytic tumors and use these patients as a validation dataset.

### Independent validation glioma datasets

#### The Glioma Longitudinal AnalySiS (GLASS) data

We used the Synapse API to download copy number segmentation files (variants_gatk_seg) for 201 primary diffuse astrocytic glioma in the Glioma Longitudinal AnalySiS (GLASS) dataset (Data Release version 2019-03-28) [6, 18]. We processed these data with GISTIC with the parameters described below. This patient cohort consisted of 143 IDH-wildtype glioblastomas and 58 IDH-mutant astrocytomas determined from available IDH mutation annotations. We did not use data from GLASS oligodendroglioma patients because there were too few. Patients’ clinical variables including overall survival (N = 184) and age (N = 186) were also downloaded.

#### Jonsson et al. data

We downloaded copy number segmentation files from cBioPortal [13, 24] for 432 primary diffuse glioma patients originally described in Jonsson et al. [31] and processed them with GISTIC as described below. Labels for IDH mutational status and 1p/19q-codeletion status were determined from criteria in the fourth edition of the WHO classification of CNS tumors. This patient cohort consisted of 319 IDH-wildtype gliomas, 63 IDH-mutant astrocytomas, and 50 oligodendrogliomas. Copy number alteration data were derived from targeted sequencing (MSK-IMPACT or FoundationOne) as described in [31]. Published clinical variables, including overall survival (N = 432) and age (N = 432) for all patients, were also downloaded.

#### Capper et al.

Illumina 450 k methylation IDAT files for 489 non-recurrent diffuse gliomas made available by the authors of Capper et al. [10] were downloaded from the NCBI Gene Expression Omnibus (GEO) under accession number GSE109381. These methylation data were processed into copy number segmentation files using the R packages *minfi* [5] and *conumee* [29], and copy number calls were computed from these segmentation files using GISTIC as described below. This patient cohort consisted of 298 IDH-wildtype glioblastomas, 110 IDH-mutant astrocytomas, and 81 IDH-mutant and 1p/19q-codeleted oligodendrogliomas as determined by criteria in the fourth edition of the WHO classification of CNS tumors. Methylation-based molecular subtype labels from version 11 of the random forest classifier released by Capper et al. were also downloaded. Patient age (N = 420) was determined from published clinical variables, but outcome data was not available.

### REMBRANDT prediction glioma dataset

Binary CN4.cnchp files from Affymetrix Human Mapping 50 K Hind240 (N = 240) and 50 K Xba240 SNP arrays (N = 192) for 275 samples were downloaded from the REMBRANDT Database (GEO Data Set GSE108475) [28]. Affymetrix Power Tools (http://www.affymetrix.com/partners_programs/programs/developer/tools/powertools.affx February 2021, date last accessed) was used to convert the CN4.cnchp files into text files. Precomputed copy number and loss of heterozygosity analysis results from the CN4 algorithm were extracted from these files, and HmmMedianLog2Ratio values were used to estimate the underlying DNA copy number variation using the Bioconductor package *DNAcopy* [54]. GISTIC was then applied to calculate gene-level gains and losses. In general, we found that data produced by Hind SNP arrays were cleaner than those generated by Xba SNP arrays and thus we used Hind-derived SCNA data for patients who had both Hind and Xba data (N = 157). Clinical variables including overall survival (N = 220) and age (N = 208) were also downloaded. Patient age ranges (i.e., 70–74) were replaced by their median age (i.e., 72).

### GISTIC 2.0 parameters

GISTIC 2.0 (GISTIC) analysis was computed in the same manner across all datasets we generated from segmentation files [46]. GISTIC was run with the following parameters: Amplification Threshold = 0.1; Deletion Threshold = 0.1; Cap Values = 1.5; Broad Length Cutoff = 0.7; Remove X Chromosome = 0; Confidence Level = 0.99; Join Segment Size = 4; Arm Level Peel Off = 1; Maximum Sample Segments = 2,000; Gene GISTIC = 1; Q-value Threshold = 0.25; Savegene = 1; Run Broad Analysis = 1; Collapse Method = extreme. The GISTIC default hg19 reference was used for hg19 alignment; the hg38.UCSC.add_miR.160920.refgene.mat file was used for alignment to hg38. All SCNA datasets other than a version of TCGA SCNA data downloaded from the GDC Data Commons (GDC hg38) were aligned to hg19.

### Machine learning methods

We first formatted our SCNA data so that our model was robust to data acquired from older cytogenetic array technologies as well as variation in data processing pipelines. Downsampling SCNA data to chromosome arm-level resolution provided an effective solution, because such processing created a data representation that did not depend on precise gene location or GISTIC output dimension. To downsample SCNA data processed by GISTIC, we first considered amplifications (GISTIC score 2) as gains (GISTIC score 1) and homozygous deletions (GISTIC score − 2) as single copy deletions (GISTIC score − 1) so that all scores were between − 1 and 1. Because all data is in the same range, we did not perform additional normalization. Next, we assigned each chromosome arm the mean value of the set of GISTIC scores that corresponded to that chromosome arm’s genes. We ignored chromosomes X and Y as well as chromosome arms 13p, 14p, 15p, 21p, and 22p, because they had a low gene count and therefore were sensitive to noise. The final model input format was a 39-dimensional chromosome arm-level SCNA data representation. To justify this data representation, we also compared it to a 50-dimensional PCA-reduced representation of gene-level SCNA data and a 50-dimensional PCA-reduced representation of averaged cytoband-level input.

Our system consists of two stages: the first phase filters out oligodendrogliomas by screening for 1p/19q-codeletions, and the second phase passes the remaining diffuse astrocytic tumors though a binary IDH-mutation classifier. We trained and evaluated a host of machine learning classifiers that predicted IDH-mutations in adult diffuse astrocytic glioma. These classifiers were implemented using the Python packages *scikit-learn* [50]. We used the Python package *PyCaret* [2] to prototype, tune, and calibrate our models. Area under the receiver operating characteristic curve (AUC) was maximized during model tuning. Random hyperparameter searches were conducted via tenfold cross-validation within the cross-validation loop for cross-validated results, and on the entire training set for predictions on the four held-out validation sets. We report results from L2-penalized logistic regression, random forest, multilayer perception, and support vector machine with a radial kernel, as well as an ensemble of these models. All cross-validated results are reported as the average of 1000 cross-validation trials. All hyperparameter choices, including model class, were made during cross-validation and only one model was applied to the independent validation datasets and the holdout TCGA validation set. All UMAP (Uniform Manifold Approximation and Projection) [44] embeddings were generated from gene-level GISTIC scores using 15 nearest neighbors and the Manhattan distance metric.

### Interpretability

We used SHapley Additive exPlanations (SHAP), as implemented in the Python package *shap* [42], to interpret our patient-level model predictions. The SHAP algorithm assigns a value to each feature used to represent a patient that indicates how responsible that feature is to the prediction the model gives to that specific patient. The SHAP algorithm also considers all features at once, rather than independently, and thus captures feature interactions. To get patient-specific SHAP values for cross-validated results, we tracked each model that correctly or incorrectly classifies each sample over 1000 cross-validation trials. Furthermore, because each model was calibrated, we had to average the SHAP value for each uncalibrated base estimator for each of 10 calibration folds per model before averaging these values across all cross-validation trials in which the sample was correctly classified and all of those in which the sample was incorrectly classified. This process led to two SHAP values for each feature for each patient: the average SHAP value for the cross-validation trials during which the patient was misclassified and the average SHAP value for cross-validation trials during which the patient was correctly classified.

### Statistical analysis

We evaluated the performance of our models with the following metrics: area under the receiver operating characteristic curve (AUC), balanced accuracy (bal accuracy), F1 score, precision, recall, and Mathews correlation coefficient (MCC). Their definitions are given below in terms of true positive (TP), true negative (TN), false positive (FP), and false negative (FN) predictions.

$$F1= \frac{TP}{TP+ \frac{1}{2}(FP+FN)}, \mathrm{Precision}= \frac{TP}{TP+FP}, \mathrm{Recall}= \frac{TP}{TP+FN}$$

$$\mathrm{Balanced\,Accuracy}= \frac{1}{2}\left(\frac{TP}{TP+FN}+\frac{TN}{TN+FP}\right)$$

$$\mathrm{MCC}= \frac{TP\cdot TN-FP\cdot FN}{\sqrt{(TP+FP)(TP+FN)(TN+FP)(TN+FN)}}$$

We focused primarily on the AUC and MCC scores. We preferred MCC over F1, accuracy, precision, and recall because MCC does not depend on which class we designate as positive and because MCC considers all four prediction categories (TP, FP, TN, FN) and their magnitudes [14]. We optimized for high AUC rather than MCC during model training, because AUC gives a better measure of model robustness.

## Results

### Overview of global SCNAs in adult diffuse gliomas

**Fig. 1:**
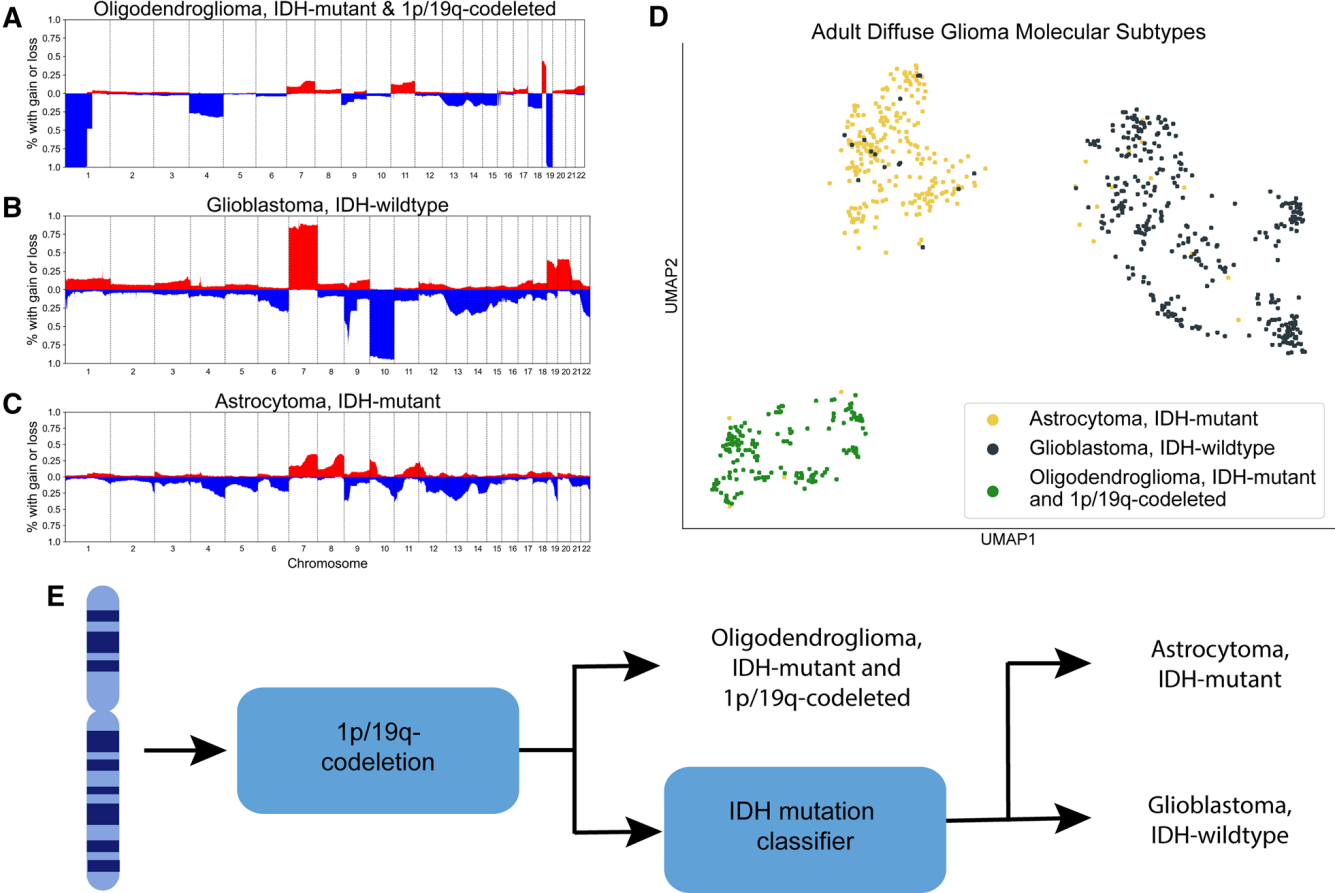
Somatic copy number alteration (SCNA) profiles of adult diffuse glioma molecular subtypes in the TCGA. **A**–**C** Copy number frequency plots showing SCNA profiles for each of the three dominate molecular subtypes of adult diffuse glioma. **D** UMAP landscape of SCNA data highlighting the separation of the major molecular subtypes based solely on global copy number information. **E** Overview schematic of our approach to use SCNA profiles to predict IDH status in diffuse gliomas

SCNA data is a promising predictor of adult diffuse glioma molecular subtype, because each adult diffuse glioma subtype exhibits a distinctive DNA structure. Oligodendrogliomas are characterized by the presence of an IDH mutation and a 1p/19q-codeletion (Fig. 1A). Adult-type IDH-wildtype diffuse gliomas predominantly display simultaneous gain of whole chromosome 7 and loss of whole chromosome 10 (+ 7/ − 10) (Fig. 1B). IDH-mutant astrocytomas have comparatively fewer large-scale SCNAs (Fig. 1C). Furthermore, unsupervised methods have shown that these subtypes are largely separable by SCNA data even in low dimensions (Fig. 1D) [7, 16, 17]. This indicates that a supervised system can robustly predict patient adult diffuse glioma molecular subtype from tumor SCNA data.

SCNA-based models can also incorporate domain knowledge regarding glioma subtype DNA structure, which would otherwise be difficult to learn in a purely data-driven manner. All oligodendrogliomas harbor translocation-mediated 1p/19q-codeletions that result in single-copy loss of chromosome arms 1p and 19q and intact status of chromosome arms 1q and 19p [30]. Because 1p/19q-codeletions are directly computable from SCNA data, we proposed a two-stage classification system for the prediction of adult diffuse glioma molecular subtype in which 1p/19q-codeleted oligodendrogliomas are identified in the first phase and the remaining diffuse astrocytic tumors are passed through a binary IDH mutation classifier in the second phase (Fig. 1D). Additionally, we computed + 7/ − 10 to verify that all tumors in our TCGA training were adult diffuse gliomas. Histological lower-grade IDH-wildtype diffuse gliomas without confirmed + 7/ − 10, *EGFR* amplification, or *TERT* promoter (*TERT*p) mutation are considered to be pediatric-type diffuse gliomas and are genetically and biologically distinct from adult diffuse gliomas, and thus less relevant to our current study [8, 40, 41].

### Determining thresholds for 1p/19q-codeletion

**Fig. 2:**
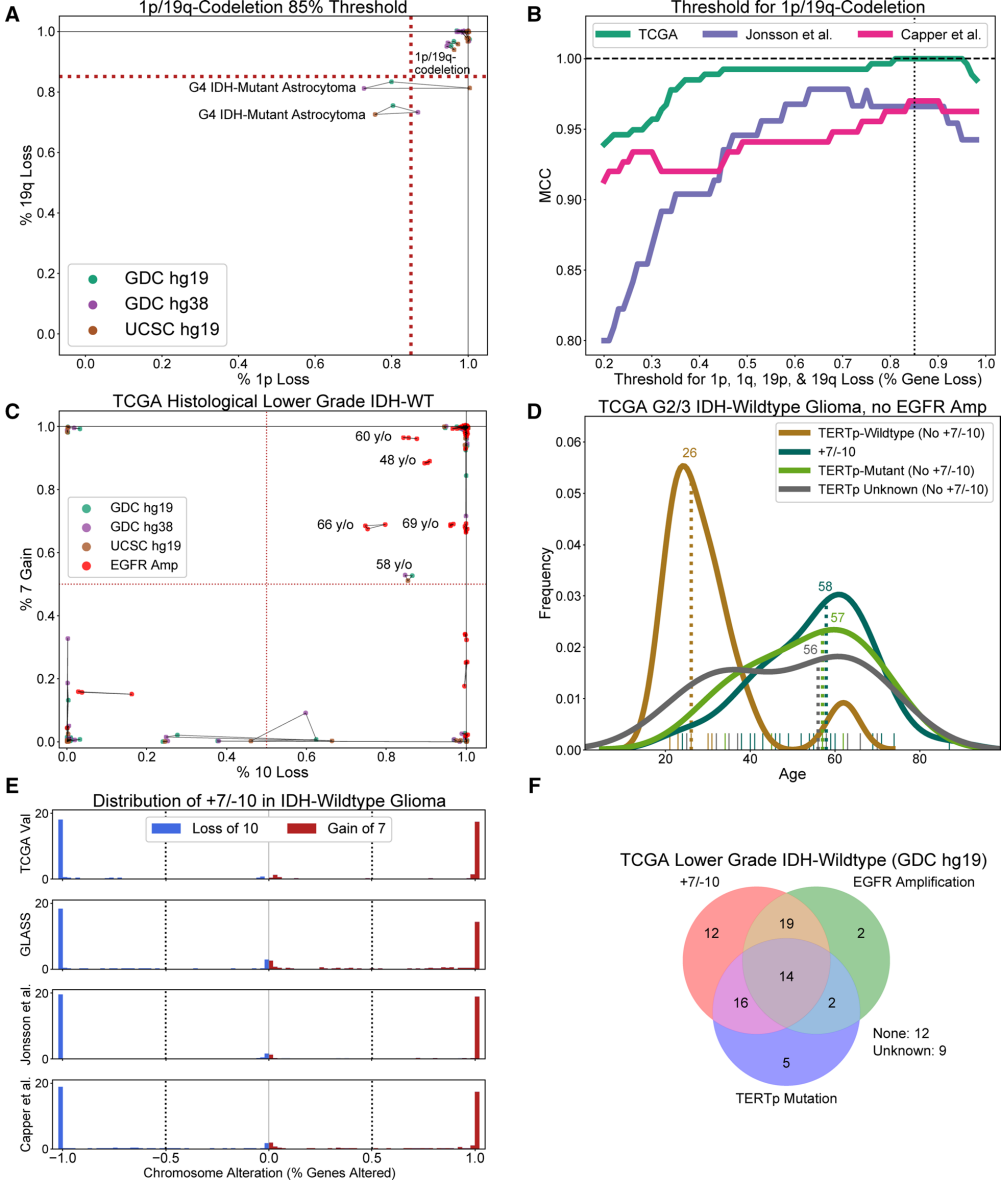
Determining rational thresholds for establishing 1p/19q-codeletion and + 7/ − 10 status. **A** A scatter plot showing the percentage of genes lost on chromosome arm 1p against the percentage of genes lost on 19q for all tumors with all of three TCGA SCNA pipelines showing at least 50% gene loss on 1p and 19q and at most 85% genes loss on 1q and 19p. All tumors that satisfy an 85% threshold on all four chromosome arms are predicted to be 1p/19q-codeleted oligodendrogliomas; all those that do not are predicted to be IDH-mutant astrocytomas. Lines connect points that represent the same patient. **B** An 85% threshold for 1p/19q-codeletions is optimal for the TCGA training set and an independent validation dataset published by Capper et al. as measured by MCC. It is nearly optimal on an independent validation dataset published by Jonsson et al. **C** A scatter plot of percent gain/loss of genes on chromosomes 7 and 10, respectively, for 91 histological lower-grade TCGA IDH-wildtype gliomas. All those without *EGFR* amplification and at least 10% gene gain/loss on chromosomes 7 and 10 also satisfy a 50% threshold. **D** Histological lower grade TCGA IDH-wildtype gliomas without *EGFR* amplification who do not meet a 50% threshold for + 7/ − 10 are much younger than those who do. This age difference increases when *TERT*p mutation status is accounted for. **E** A 50% threshold for + 7/ − 10 separates + 7 and − 10 from intact 7 and intact 10 across three independent validation sets and the TCGA holdout validation set. **F** Of all TCGA histological lower-grade IDH-wildtype diffuse gliomas, 21 do not or cannot be confirmed to meet the WHO 2021 criteria for adult diffuse glioma

Calling 1p/19q-codeletions from gene-level SCNA data requires setting a threshold for the proportion of genes lost on chromosome arms 1p and 19q and the proportion of genes retained on chromosome arms 1q and 19p. To establish such a threshold, we considered TCGA SCNA data derived from three different pipelines (UCSC hg19, GDC hg19, GDC hg38) to account for variation in data processing. For each pipeline, we observed that an 85% threshold for gene loss on chromosome arms 1p, 1q, 19p, and 19q separated all oligodendrogliomas (N = 171) from all astrocytic tumors (N = 615) in our TCGA training set, including several IDH-mutant astrocytomas that would have been considered 1p/19q-codeleted oligodendrogliomas using slightly lower thresholds (Fig. 2A, Additional file 2: Fig. 2A). This 85% threshold was also optimal or nearly optimal for two independent validation sets containing at least 50 1p/19q-codeleted oligodendrogliomas published by Capper et al. [10] (MCC = 0.97) and Jonsson et al. [31] (MCC = 0.97), respectively (Fig. 2B). Between these two validation sets, only one (0.1%) astrocytic tumor was predicted to be 1p/19q-codeleted (Additional file 2: Fig. 2B, C). Although six (4.6%) labeled oligodendrogliomas were predicted to be astrocytic tumors, four of the six misclassified oligodendrogliomas harbored monosomy of chromosome 1 and/or monosomy of chromosome 19, which is inconsistent with the unbalanced translocation mechanism associated with the development of oligodendrogliomas (Additional file 2: Fig. 2B, D) [27, 30]. Therefore, these were not model errors. The two other misclassified oligodendrogliomas fail to meet our 85% threshold, because one sample lost only 75% of 1p and the other only 71% of 19q. Regardless, we did not lower our threshold to include these patients, because lower thresholds risked misclassifying more IDH-mutant astrocytomas as 1p/19q-codeleted oligodendrogliomas. To assess the robustness of our 1p/19q-codeletion screen to genome coverage, this screen was applied to ULP-WGS derived TCGA SCNA data available for patients in the TCGA training set (556 astrocytic tumors, 169 oligodendrogliomas). Only one tumor was misclassified (MCC = 0.996), suggesting that even ultra-low coverage (0.1x) is sufficient for accurate identification of 1p/19q-codeletions. All 1p/19q-codeletion predictions are given in the Additional file 1: Table 4.

### Determining thresholds for + 7/ − 10

Knowledge of + 7/ − 10 status was required to verify that all histologically-defined WHO grades 2 and 3 IDH-wildtype gliomas in our TCGA training set were adult-type diffuse gliomas [40, 41]. To ascertain + 7/ − 10 status, we determined a threshold for the proportion of genes necessary to determine aneuploidy. This was done by identifying all TCGA patients whose adult glioma verification depended on their + 7/ − 10 status and settling on the highest threshold for + 7/ − 10 that did not exclude tumors whose age and outcome were consistent with quintessential IDH-wildtype glioblastoma. A 50% threshold for + 7/ − 10 was the highest threshold capable of calling a 58-year-old short-term survivor (OS = 13 months) without *EGFR* amplification an adult diffuse glioma (Additional file 2: Fig. 3A). Given that the next lowest threshold that would have changed a TCGA patient's adult glioma status was below 10%, we adopted 50% as our threshold for determining + 7/ − 10 status (Fig. 2C). As desired, this threshold divided histological lower-grade IDH-wildtype gliomas without *EGFR* amplification into two groups with significantly different age distributions (median age 35 vs. 59.5, *p* < 0.0005, Mann–Whitney U-test), especially when *TERT*p mutation status was accounted for (median age 26) (Fig. 2D, Additional file 2: Fig. 3B, C). Furthermore, a 50% threshold separated the bimodal distributions of + 7 and − 10 for IDH-wildtype diffuse gliomas of all histological grades in three independent validation sets in addition to the TCGA validation set (Fig. 2E). Finally, applying this 50% threshold to our TCGA training data, we excluded 21 TCGA patients: 12 lacked + 7/ − 10, *EGFR* amplification, and *TERT*p mutation and 9 lacked *EGFR* amplification and + 7/ − 10 and had unknown *TERT*p mutation status (Fig. 2F, Additional file 2: Fig. 3D).

### IDH mutation classifier design

#### Justification of design decisions

**Fig. 3:**
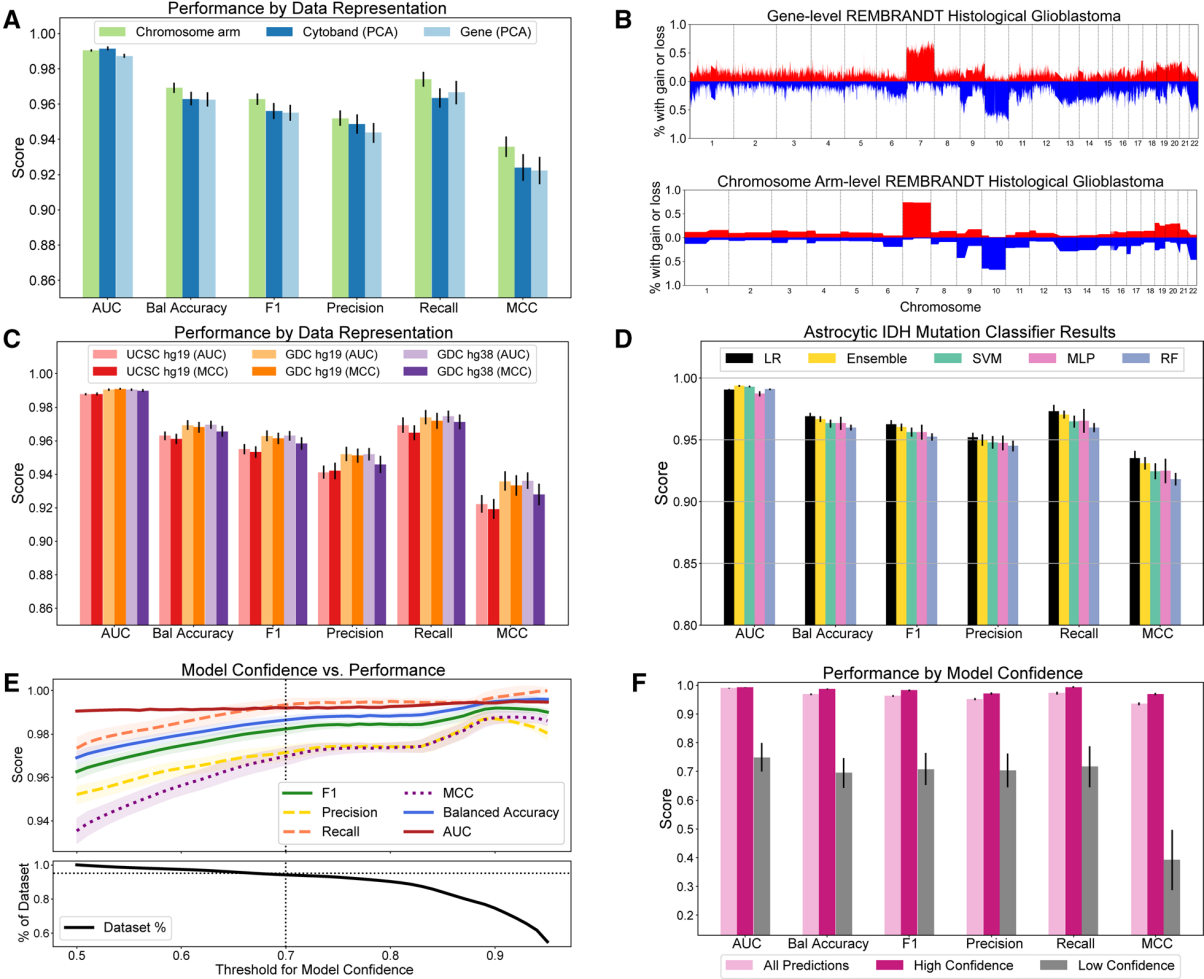
Cross-validated IDH mutation classifier development. **A** Cross-validated results showed that IDH mutation logistic regression classifiers trained on chromosome arm resolution SCNA data performed better than classifiers trained on PCA reduced gene-level or PCA reduced cytoband-level SCNA data. **B** Downsampling gene-level SCNA data to chromosome arm resolution smoothed noisy SCNA data derived from older cytogenetic arrays. **C** A logistic regression model trained on TCGA SCNA data aligned to hg19 and optimized for maximizing the AUC score performed better than other parameter choices. **D** Logistic regression mostly outperformed other model classes, including an ensemble of all listed models, across five metrics. **E** Our model performance increased monotonically when restricted to samples of increasing prediction confidence. This indicated that the calibration of our model’s output probabilities was effective. Standard deviation values for each metric over 1000 cross-validation trials are shaded in. **F** Restricting model predictions to those made with confidence greater than 0.7 greatly increased model performance

The 1p/19q-codeletion screen that comprises the first phase of our adult diffuse glioma molecular subtype predictive system passes predicted adult astrocytic gliomas to the system’s second phase IDH mutation classifier. To build this classifier, we trained a logistic regression (LR) model, optimized to maximize AUC, on hg19-aligned SCNA data (GDC hg19) downsampled to chromosome arm-level resolution. The choices made during model development were justified by cross-validated experiments on the 615 adult astrocytic gliomas in our TCGA training set. Downsampled chromosome arm-level SCNA data performed as well or better than other low-dimensional SCNA input representations including PCA reduced SCNA data (Fig. 3A) and were more interpretable and similarly robust to noise. Indeed, downsampling to chromosome arm-level resolution had a pronounced smoothing effect on SCNA data obtained from older cytogenetic array technologies, such as the REMBRANDT study SCNA data (Fig. 3B) [28]. We trained our model to maximize AUC performance during its parameter search and used the GDC hg19 version of the TCGA SCNA data for our training samples, because this combination of metric optimizer and dataset yielded the best cross-validated results (Fig. 3C). We selected logistic regression as our model class, because our LR model outperformed (AUC = 0.990 ± 0.001, MCC = 0.935 ± 0.006) a suit of other machine learning classifiers across nearly all evaluation metrics (Fig. 3D). All predictions are given in the Additional file 1: Table 5.

#### Model calibration facilitates the rejection of inaccurate predictions

To recognize patients whose model prediction may not be reliable, such as patients whose tumor is not an adult-type diffuse glioma, we calibrated our model’s output probabilities and gave the option to reject low-confidence predictions. Such calibration forced the model’s output probabilities to better represent prediction confidence, which should be low when the model evaluates tumors that do not resemble tumors in its training set. As desired, we saw that our model performed better across all metrics on patients whose prediction confidence was above 70% (AUC = 0.992 ± 0.001, MCC = 0.970 ± 0.004) compared to patients with lower confidence predictions (AUC = 0.75 ± 0.05, MCC = 0.39 ± 0.1), despite excluding only 5% of the dataset (Fig. 3E, F).

### Interpretation of IDH mutation classifier cross-validation predictions

#### Misclassified patients were rare and involved + 7/ − 10

**Fig. 4:**
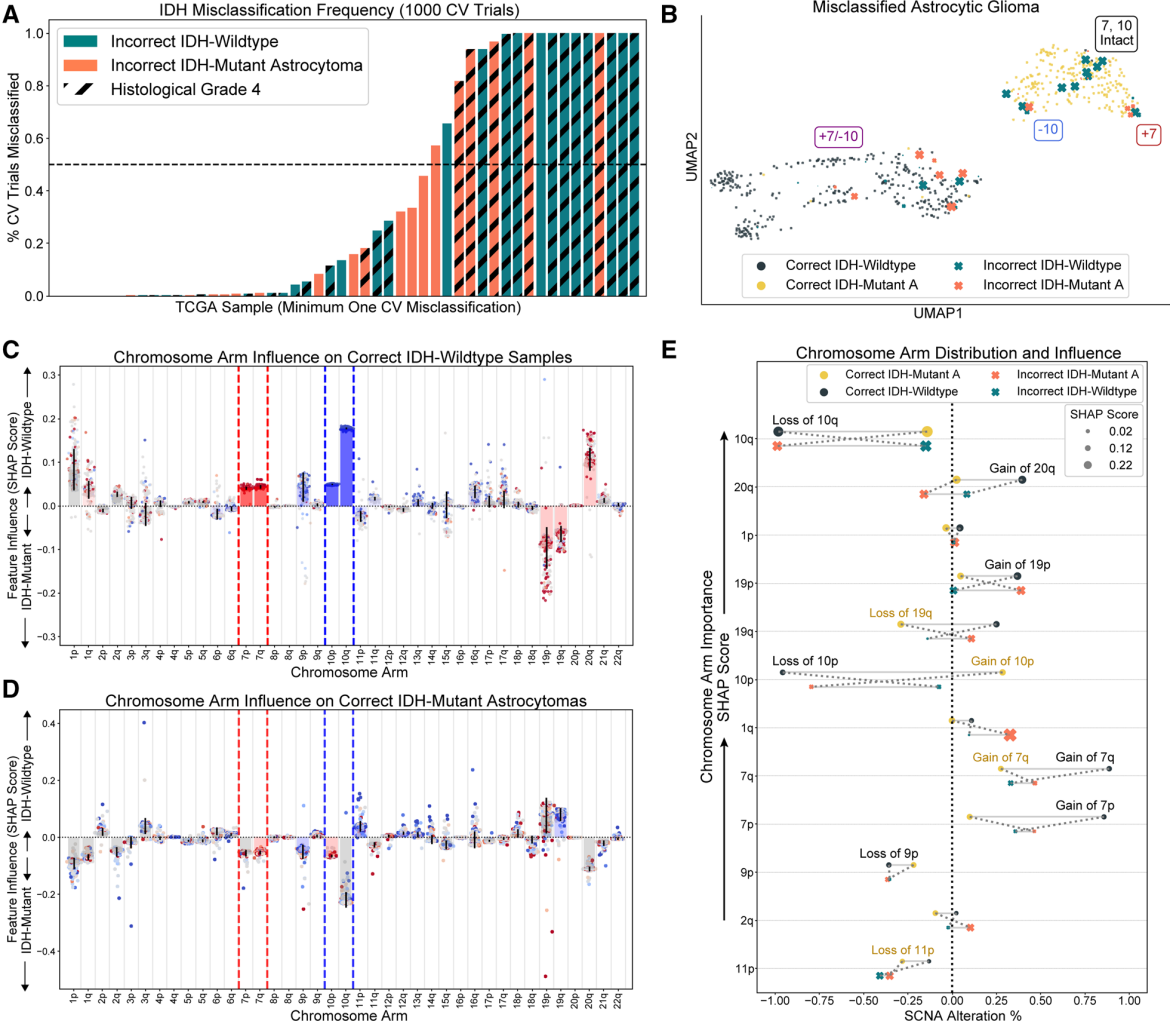
Interpretation of cross-validated results. **A** All gliomas in the TCGA training set that were misclassified at least once over 1000 cross-validation trials. Only 3.4% of IDH-wildtype gliomas and 2.3% of IDH-mutant astrocytomas are misclassified in over 50% of trials. Misclassified IDH-mutant astrocytomas are disproportionately WHO grade 4. **B** Misclassified samples show regionality on a UMAP SCNA landscape: most misclassified IDH-mutant astrocytomas are found in an area of predominantly correctly classified IDH-wildtype tumors, and most misclassified IDH-mutant tumors are found in the area dominated by correctly classified IDH-mutant astrocytomas. Point-size of misclassified samples indicates the frequency of misclassification. **C** SHAP results of correctly classified IDH-wildtype tumors indicate that chromosome arm 10q drove correctly classified IDH-wildtype predictions. Each patient is assigned a point per chromosome arm. The magnitude of each point’s vertical coordinate is an indication of how influential the average SCNA status of the chromosome arm was to the classifier’s prediction. Positive vertical values favor IDH-wildtype predictions; negative values indicate a preference for IDH-mutant astrocytoma predictions. The color of each point corresponds to the average chromosome arm SCNA value: blue indicates loss, and red indicates gain. The magnitude of the bars drawn for each chromosome arm is the average chromosome arm SHAP score for all patients plotted; the bar’s color indicates the average SCNA state across all patients plotted. **D** SHAP results of correctly classified IDH-mutant astrocytomas; intact 10q drives these predictions. **E** A visualization of average SHAP score and average chromosome arm SCNA status over all correct and incorrect model predictions of IDH-wildtype and IDH-mutant astrocytomas shows the top 12 chromosome arms’ influence on model predictions. The height of groups of connected points orders each chromosome arm’s average influence (SHAP score) on correct predictions. The x-axis indicates the average extend of SCNA loss or gain of each chromosome-arm for the cohort of patients each point represents. The size of each point represents the average influence the chromosome arm has. Solid lines connect correct or incorrect predictions; dotted lines connect groups of the same IDH-subtype. Crossing dotted lines identify chromosome arms whose SCNA profile mimics the opposite IDH subtype when mistaken by our model

Given the robustness of our IDH mutation classifier, we were particularly interested in the rare patients it misclassified. Across 1000 cross-validation trials, 3.4% (N = 12) of IDH-wildtype glioblastomas and 2.3% (N = 6) of IDH-mutant astrocytomas were misclassified in at least 50% of trials (Fig. 4A). Misclassified IDH-mutant astrocytomas had increased copy number burden, especially on chromosome 10, were disproportionately WHO grade 4 (*p* < 0.0001, Fisher’s Exact), and followed a clinical course significantly worse than correctly classified IDH-mutant astrocytomas (OS = 2.8 vs 7.3 years, HR = 1.97, *p* < 0.001, log-rank) (Additional file 2: Fig. 4A, B, C). Most misclassified IDH-mutant astrocytomas were embedded in a cluster of IDH-wildtype tumors defined by + 7/ − 10 on a UMAP SCNA landscape, indicating that the DNA structure of these tumors resembles that of IDH-wildtype glioblastomas (Fig. 4B). On the other hand, misclassified IDH-wildtype glioblastomas tended to have fewer SCNAs than their correctly classified counterparts, especially on chromosomes 7 and 10, and primarily inhabited a region on the UMAP landscape occupied by IDH-mutant astrocytomas (Additional file 2: Fig. 4D). Unlike IDH-mutant astrocytomas, however, no difference in histological grade or patient outcome between correctly and incorrectly classified IDH-wildtype glioblastomas was observed (Additional file 2: Fig. 4E, F).

#### Chromosome arm 10q holds greatest influence over IDH mutation classification

The SHapley Additive exPlanations (SHAP) algorithm assigns a value to each chromosome arm for each patient that indicates how responsible that chromosome arm’s SCNA status is to the prediction the model gives that patient [42]. A SHAP analysis of our IDH mutation classifier’s cross-validated predictions indicated that loss of chromosome arm 10q drove IDH-wildtype predictions and that gain of chromosome arms 20q and 1p were more discriminative features than 10p loss or 7p and 7q gain (Fig. 4C). Similarly, intact or marginal loss of 10q, intact 20q, intact chromosome 1, and marginal gains of 10p drove correct IDH-mutant astrocytoma predictions (Fig. 4D). Unsurprisingly, intact 10q in IDH-wildtype gliomas or loss of 10q in IDH-mutant astrocytomas were the primary drivers of astrocytic tumor misclassification (Additional file 2: Fig. 5). In general, our model’s mistakes were intuitive: a small subset of tumors from each subtype exhibited SCNA patterns more consistent with the opposite subtype on chromosome arms weighed heavily by the model, especially chromosome arm 10q (Fig. 4E).

**Fig. 5:**
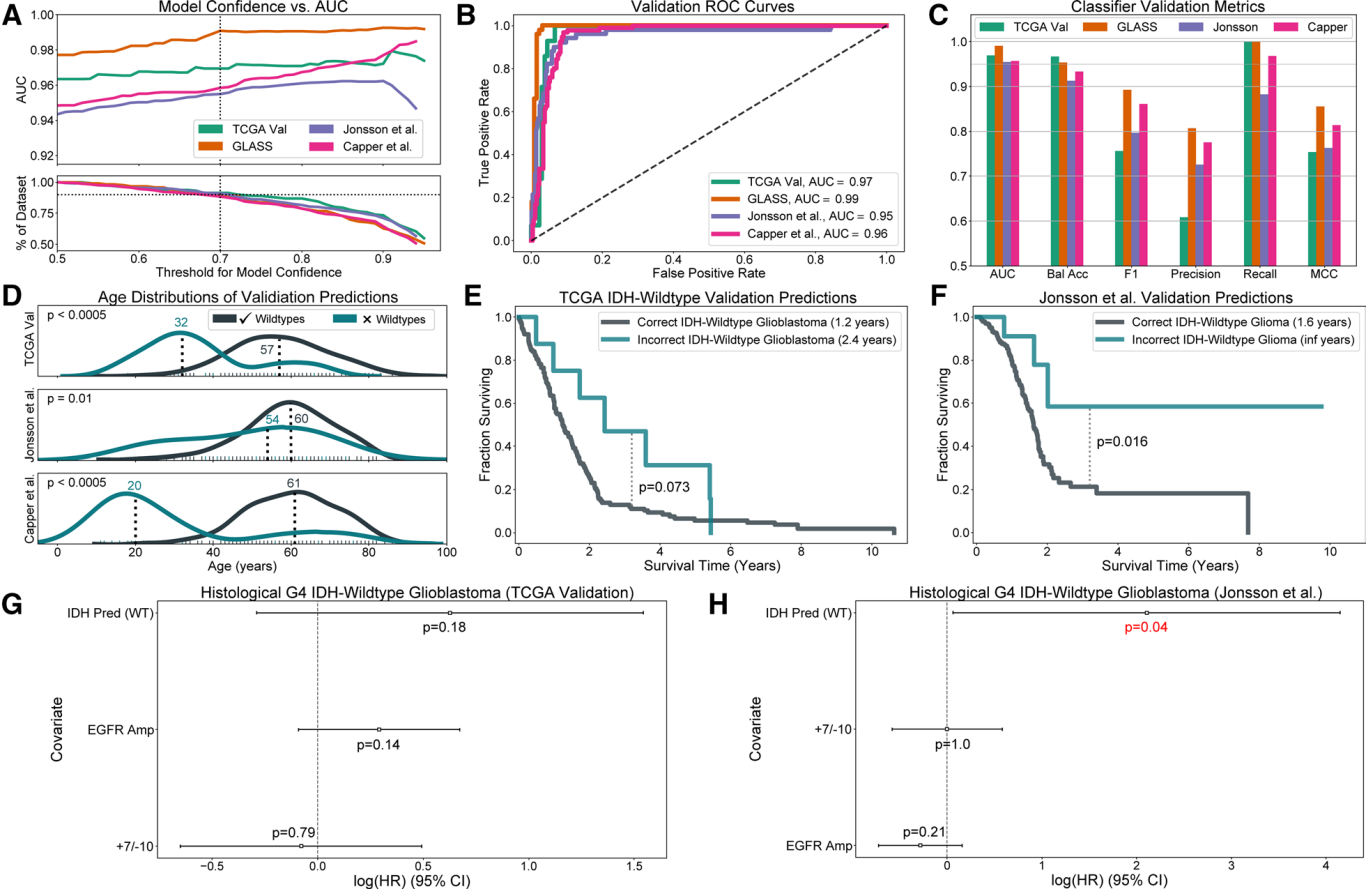
Validation results for four independent glioma datasets. **a** The IDH mutation classifier’s performance increased with prediction confidence on four validation sets, confirming the utility of model calibration. **b** ROC curves show that our LR model performs well on four separate validation sets. **c** Besides AUC, our model performs well four additional metrics. Recall is significantly higher than precision across validation sets, indicating that the model performs better on IDH-mutant astrocytomas than IDH-wildtype tumors. **L** Misclassified IDH-wildtype tumors are significantly younger than their correctly classified counterparts in three validation datasets (Mann–Whitney U). **e, f** Misclassified IDH-wildtype tumors in the TCGA validation set and a dataset published by Jonsson et al. tend to have better outcomes than correctly classified IDH-wildtype diffuse gliomas. **g, h** Plots of the results of two Cox proportional hazard models of histological WHO grade 4 IDH-wildtype glioblastomas that incorporated our model’s IDH mutation prediction, *EGFR* amplification, and + 7/ − 10

### Validation of the IDH mutation classifier on three independent datasets and the TCGA validation dataset

#### Validation results

Our IDH mutation classifier performed well across three independent validation datasets and our holdout TCGA cohort of histological grade 4 patients with surrogate IDH labels not found from IDH sequencing. As observed during cross-validation, model performance increased with prediction confidence (Fig. 5A, Additional file 2: Fig. 6). When evaluated on patients with model confidence greater than 70%, our IDH mutation classifier achieved AUC scores greater than 0.95 on each dataset (Fig. 5B). Recall was substantially higher than precision, indicating that the model performed better on IDH-mutant astrocytomas than IDH-wildtype gliomas (Fig. 5C). Our IDH mutation classifier also performed well (AUC = 0.98) on a version of our TCGA validation datasets whose SNCA data was derived from ULP-WGS data, indicating that this model is robust to ultra-low genome coverage (Additional file 2: Fig. 7). Results for model predictions on all patients with diffuse astrocytic gliomas are given in the Additional file 1: Table 6 and Additional file 2: Fig. 8.

**Fig. 6:**
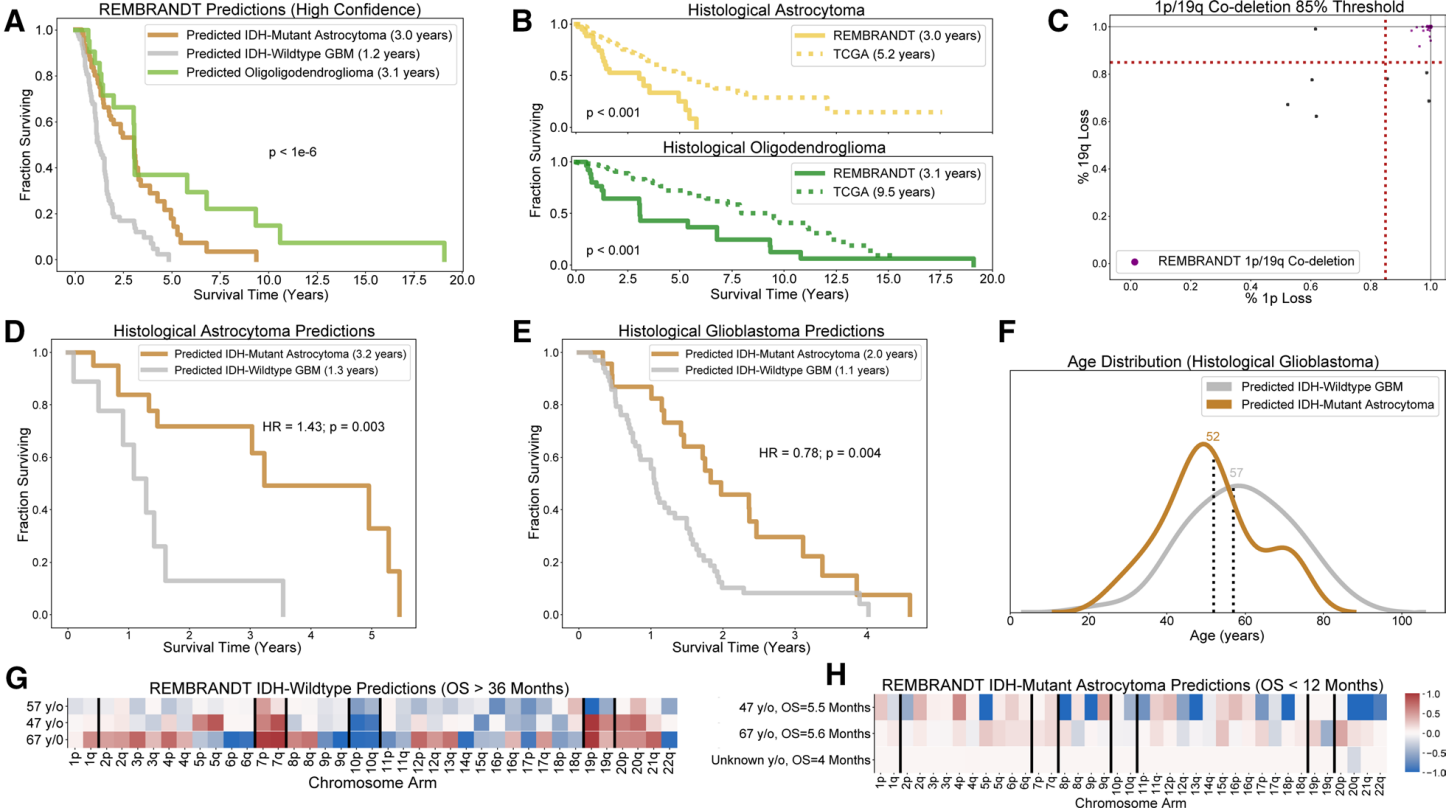
Adult diffuse glioma molecular subtype predictions for the REMBRANDT study. **A** Predictions for all REMBRANDT patients show typical median survival for IDH-wildtype glioblastoma but worse outcomes for predicted IDH-mutant astrocytomas and oligodendrogliomas compared to those in the TCGA. **B** Unlike histologically-defined glioblastomas, histological astrocytomas and oligodendrogliomas in the REMBRANDT fair significantly worse than TCGA histological astrocytomas and oligodendrogliomas. **C** An 85% threshold for chromosomes 1p, 1q, 19p, and 19q successfully captures the densest area of patients who pass a 50% threshold for 1p/19q-codeletion. **D** Within the cohort of REMBRANDT histological astrocytomas, predicted IDH-wildtype tumors fair significantly worse than predicted IDH-mutant astrocytomas. **E** Within the cohort of REMBRANDT histological glioblastomas, predicted IDH-wildtype tumors’ outcomes are significantly worse than predicted IDH-mutant astrocytomas. **F** Predicted IDH-wildtype tumors do not show a subpopulation of young patients that would indicate the presence of pediatric-type tumors or misclassified IDH-mutant astrocytomas. **G** SCNA profiles of predicted IDH-wildtype tumors that live longer than 3 years. Three tumors show + 7/ − 10, of which two show co-gain of chromosomes 19 and 20, and are likely IDH-wildtype tumors. The remaining tumor shows mild losses on chromosome 10. **H** SCNA profiles of predicted IDH-mutant astrocytoma that live less than 12 months. None show SCNA characteristics of IDH-wildtype glioblastoma, suggesting that they are correctly classified IDH-mutant astrocytomas or possibly IDH-wildtype glioblastomas with *TERT*p mutations

#### Misclassified IDH-wildtype diffuse gliomas were younger and lived longer

Misclassified IDH-wildtype tumors were significantly younger than correctly classified IDH-wildtype tumors in our TCGA validation set (*p* < 0.001, Mann–Whitney U test) and validation sets published by Jonsson et al. (*p* < 0.01, Mann–Whitney U test) and Capper et al. (*p* < 0.001, Mann–Whitney U test) (Fig. 5D). On two validation sets that had patient outcome data, patients with IDH-wildtype gliomas that were predicted to be IDH-mutant astrocytomas demonstrated significant (*p* = 0.016, Jonsson et al., log-rank) or marginally significant (*p* = 0.073, TCGA validation, log-rank) improvement in survival compared to correctly predicted IDH-wildtype gliomas (Fig. 5E, F). Interestingly, these results remained significant when restricted to histological WHO grade 4 tumors (Additional file 2: Fig. 9). No consistent age or survival difference was observed in IDH-mutant astrocytomas (Additional file 2: Fig. 10). Likewise, the same IDH-wildtype glioma age and outcome associations observed in the other validation sets did not hold in the GLASS, likely due to patient inclusion bias, which is inherent in datasets made up of tumors known to have second resections (Additional file 2: Fig. 11) [16].

Because our model’s misclassified IDH-wildtype patients were associated with the absence of + 7/ − 10 and younger age, attributes of pediatric-type tumors, we tested whether the status cIMPACT-NOW update 3 [8] molecular markers (which are incorporated in the upcoming WHO CNS tumor classification fifth edition [40]) explained the improved survival of misclassified histological WHO grade 4 IDH-wildtype tumors. The status of + 7/ − 10 and *EGFR* amplification, but not *TERT*p mutation, were used in multivariate Cox proportional hazard models [19] because too few *TERT*p mutation labels were available. In these analyses, the hazard ratio of the model’s IDH mutation prediction was higher than either that of + 7/ − 10 or *EGFR* amplification in the TCGA validation set and Jonsson et al. dataset, although the confidence intervals varied (Fig. 5G, H). This evidence indicated that the observed survival benefit of misclassified histological grade 4 IDH-wildtype glioblastoma was due more to the model’s prediction than + 7/ − 10 or *EGFR* amplification status. Complementary univariate tests of combinations of + 7/ − 10 and *EGFR* amplification in the TCGA validation set and the Jonsson et al. validation set showed inconsistent prognostic utility of these markers among histological grade 4 IDH-wildtype glioblastoma (Additional file 2: Fig. 12). For example, *EGFR* application was not prognostic in the Jonsson et al. dataset, and the absence of + 7/ − 10 did not convey significant survival benefit in the TCGA validation set. With limited *TERT*p mutation status information, the clinical utility of the cIMPACT-NOW update 3 molecular markers cannot be fully assessed, but the improved survival of misclassified histological WHO grade 4 IDH-wildtype tumors is unlikely a reflection of these molecular markers as we measure them.

#### Many misclassified IDH-wildtype glioblastomas harbor pediatric-type SCNA profiles

Of all misclassified histological WHO grade 4 IDH-wildtype glioblastoma patients in our four validation sets, only 19% showed IDH-wildtype glioblastoma-like SCNA features + 7/ − 10 or *EGFR* amplification (Additional file 2: Fig. 13A). While *TERT*p mutation status was unavailable, pediatric tumors, originally diagnosed as IDH-wildtype glioblastoma, were present in the cohort of misclassified IDH-wildtype glioblastomas published by Capper et al. In this dataset, 88% of misclassified IDH-wildtype samples were diffuse hemispheric glioma, H3 G34-mutant, WHO grade 4 while only 2% of correctly classified samples were diffuse hemispheric glioma, H3 G34-mutant (*p* < 1e−22, Fisher’s exact, Additional file 2: Fig. 13B). The presence of these tumors, common in adolescent and young adults, explained the age difference between correctly and incorrectly classified IDH-wildtype glioblastomas in the dataset published by Capper et al. (Additional file 2: Fig. 13C) [32].

### IDH and 1p/19q-codeletion prediction results in the REMBRANDT dataset

#### REMBRANDT predictions and outcomes comparisons

Prior to applying our system to the REMBRANDT dataset, we retrained the IDH mutation classifier on all patients in our training and validation sets other than the 41 diffuse hemispheric glioma, H3 G34-mutant, WHO grade 4 provided by Capper et al. (N = 1729). Predictions on all REMBRANDT patients with model confidence greater than 70% show that predicted IDH-wildtype glioma, regardless of tumor histological grade, have similar survival trajectories and median overall survival (OS = 1.1 years) as IDH-wildtype glioblastomas in the TCGA (OS = 1.2 years) (Fig. 6A). On the other hand, predicted IDH-mutant astrocytomas (OS = 3 years) and predicted IDH-mutant and 1p/19q-codeleted oligodendrogliomas (OS = 3.1 years) in the REMBRANDT dataset have significantly shorter median survival than TCGA IDH-mutant astrocytomas (OS = 7.3 years) and 1p/19q-codeleted oligodendroglioma (OS = 11.2 years), respectively. This discrepancy can be attributed to differences in average outcomes of histological astrocytomas (OS = 3.0 vs. 5.2 years) and histological oligodendrogliomas (OS = 3.1 vs. 9.5 years) between the REMBRANDT study and the TCGA (Fig. 6B). Predicted REMBRANDT IDH-mutant astrocytomas are also disproportionately older and higher grade than TCGA IDH-mutant astrocytomas, although the same is not true of predicted REMBRANDT and TCGA 1p/19q-codeleted oligodendrogliomas (Additional file 2: Fig. 14). Results for model predictions on all REMBRANDT patients are given in the Additional file 1: Table 7 and Additional file 2: Fig. 15.

#### REMBRANDT 1p/19q-codeletion screen highlights limitations of histological only diagnosis

Our REMBRANDT 1p/19q-codeleted oligodendroglioma screen captured the densest area of tumors with significant gene losses on chromosome arms 1p and 19q, consistent with the 1p/19q-codeletion screens on our training and validation sets (Fig. 6C). As reported elsewhere, our predictions highlight the difficulty of absolutely distinguishing astrocytomas from oligodendrogliomas based on histomorphology alone [22, 53]. Only 46% of REMBRANDT histological oligodendrogliomas harbor predicted 1p/19q-codeletions, and only 48% of REMBRADNT tumors harboring predicted 1p/19q-codeletions were diagnosed as histological oligodendroglioma (Additional file 2: Fig. 16A). Visual inspection of patient copy number profiles showed clear evidence of 1p/19q-codeletions in our predicted oligodendroglioma patients (Additional file 2: Fig. 16B). Similarly, in patients diagnosed with oligodendroglioma for whom we did not find 1p/19q-codeletions, we saw that most (5/6) tumors that lose 1p harbor monosomy chromosome 19, prohibiting a 1p/19q-codeletion (Additional file 2: Fig. 16C). The exception was a single patient with full loss of 1p and intact 1q and 19p, but whose proportion of 19q loss falls slightly below our 85% threshold (79%): this patient’s tumor was likely an oligodendroglioma (Additional file 2: Fig. 16D).

#### REMBRANDT IDH mutation classifier results on histological astrocytic tumors

Following our screen for oligodendrogliomas, we applied our IDH-mutation classifier to patients diagnosed with histological astrocytoma and glioblastoma not harboring predicted 1p/19q-codeletions. Our IDH mutation predictions on histological astrocytomas generated a dramatic survival difference between predicted IDH-wildtype gliomas and IDH-mutant astrocytomas (HR = 1.43, *p* = 0.003, log-rank) and appeared to correctly identify histological lower-grade IDH-wildtype gliomas (OS = 1.1 years) now considered to be IDH-wildtype glioblastomas (Fig. 6D). Histological glioblastomas predicted to be IDH-wildtype glioblastomas had the same median overall survival (1.1 years), and their survival trajectory was significantly worse than those of histological grade 4 tumors that were predicted to be IDH-mutant astrocytomas (HR = 0.78, *p* = 0.004, log-rank) (Fig. 6E).

#### Possible IDH mutation classifier REMBRANDT errors

To identify possible IDH mutation classification errors on the REMBRANDT dataset, we examined abnormally young or long-living predicted IDH-wildtype glioblastomas as well as abnormally old or short-living predicted IDH-mutant astrocytomas. We did not observe a subset of significantly younger patients within the cohort of predicted IDH-wildtype patients which may have represented misclassified IDH-mutant astrocytomas (Fig. 6F). Of the four predicted IDH-wildtype glioblastoma patients that lived longer than three years, two harbored hallmark + 7/ − 10 and *EGFR* amplification as well as co-gain of chromosomes 19 and 20, a documented marker for long-term survivors in IDH-wildtype glioblastoma [25] (Fig. 6G). These tumors were likely correctly classified. The remaining tumors were inconclusive. One lacked + 7/ − 10 but its patient’s age (57 years old) was consistent with IDH-wildtype glioblastoma, and the other harbored + 7/ − 10 but was atypically young (32 years old). Both lacked *EGFR* amplification. Among predicted IDH-mutant astrocytomas, we observed an older subset of patients (age range 65–80, N = 4). However, these tumors had few IDH-wildtype glioblastoma-like SCNA features: none had + 7/ − 10 and only one displayed *EGFR* amplification (Additional file 2: Fig. 17). Similarly, of the six IDH-mutant astrocytomas that lived less than 12 months, none harbored + 7/ − 10 or *EGFR* amplification, and two had nearly zero copy number alterations (Fig. 6H). These tumors were likely IDH-mutant astrocytomas, although the lack of *TERT*p mutation knowledge may have hidden IDH-wildtype glioblastomas.

## Discussion

We developed a system that predicts adult diffuse glioma molecular subtype from SCNA data, verified its accuracy on three independent validation datasets in addition to the TCGA validation dataset, and applied it to the retrospective REMBRANDT study. In a platform-independent manner, this system can robustly assign molecular subtype labels to patients with SCNA data derived from several molecular methods, including SNP-array, methylation array, whole-exome sequencing, and whole-genome sequencing. This is relevant because retrospective glioma studies with SCNA data, but no molecular subtype information, can be transformed into effective validation datasets for contemporary research. Furthermore, the gene-level thresholds we proposed for calling 1p/19q-codeletions and + 7/ − 10 are applicable to all glioma datasets with SCNA data, regardless of whether IDH information is available.

The 85% threshold we used for the 1p/19q-codeletion screen in our system’s first phase leveraged data from 786 gliomas and was validated on data from 940 tumors across two datasets. Despite the abundance of evidence supporting this threshold, we recommend visually inspecting the SCNA profiles of patients who fall near this threshold. We suspect that there exist IDH-mutant astrocytomas that lose 1p and 19q by means other than translocation and should not be considered 1p/19q-codeleted. We identified one such patient (Additional file 2: Fig. 2C) who also showed remarkably high SCNA burden. We conjecture that in this case 1p and 19q loss, along with other large-scale SCNAs, may be the result of stochastic processes. Accordingly, tumors harboring high SCNA burden that are predicted to harbor 1p/19q-codeletion by our system should be flagged for closer examination. Conversely, there are rare 1p/19q-codeleted oligodendrogliomas that do not meet our 1p/19q-codeletion threshold. Having observed two such tumors (Additional file 2: Fig. 2E), we suggest that tumors falling just shy of our 1p/19q-codeletion threshold be inspected for oligodendroglioma-like SCNA patterns outside of chromosome arms 1p and 19q. Low tumor cellularity may explain why some oligodendroglioma gliomas appear to lose significantly less than 100%, and occasionally fewer than 85%, of genes on chromosome arms 1p and 19q. It is also conceivable that IDH-wildtype tumors may meet our 1p/19q-codeletion threshold, but we saw no evidence for this.

Our threshold for + 7/ − 10 will benefit from further corroboration. A rigorous search for a threshold for + 7/ − 10 requires more histological lower-grade IDH-wildtype tumors with available *TERT*p mutation status and outcome data in addition to SCNA data. Only 12 such tumors were available in the TCGA dataset. Without *TERT*p mutation status, it is not possible to define two groups of histologically-defined WHO grades 2 or 3 IDH-wildtype glioma whose status as either pediatric-type gliomas or IDH-wildtype glioblastomas depends on the threshold set for + 7/ − 10. Therefore, it is difficult to claim a particular threshold is optimal. Instead, based on our analysis, we suggest that a 50% threshold for + 7/ − 10 is reasonable. Additional knowledge of *TERT*p mutation status is also needed for a rigorous analysis of the prognostic value of cIMPACT-NOW update 3 criteria in histological grade 4 IDH-wildtype glioblastoma. Our analysis of + 7/ − 10 and *EGFR* amplification did not consistently show a survival benefit among histological WHO grade 4 IDH-wildtype tumors that lacked these markers, but other studies have shown this difference [23]. These studies were able to identify more “triple negative” (no + 7/ − 10, no EGFR amplification, no *TERT*p mutation), primarily because their method for + 7/ − 10 determination was less sensitive–another reason to refine our threshold for + 7/ − 10. Knowledge of *TERT*p mutational status is also needed for tumor types besides IDH-wildtype glioma. For example, emerging evidence suggests that *TERT*p mutations may convey positive prognoses in IDH-mutant astrocytomas [4].

In the second phase of our system, we observed that excluding low-confidence predictions improved the classifier’s performance and may have filtered out unfamiliar tumors, such as non-adult diffuse glioma likely. However, our model calibration strategy does not reject all non-adult diffuse glioma, such as many diffuse hemispheric glioma, H3 G34-mutant in the dataset published by Capper et al. In future model iterations, we will train a multiclass classifier which will help identify other tumor types in the REMBRANDT dataset. Additional improvements include using data augmentation to generate synthetic WHO grade 4 IDH-mutant astrocytoma, especially those with losses on chromosome 10, which are underrepresented in our training set and are difficult to distinguish from IDH-wildtype glioblastoma. Additionally, ensembling our IDH mutation classifier with a model trained without chromosomes arms 7p, 7q, 10p, and 10q may improve the classification of IDH-wildtype tumors without + 7/ − 10.

We observed that misclassified histological WHO grade 4 IDH-wildtype glioblastomas tended to have better outcomes than correctly classified WHO grade 4 IDH-wildtype glioblastomas in our TCGA validation set and the dataset published by Jonsson et al. Given that these tumors were all histological WHO grade 4, the existence of pediatric-type diffuse glioma does not explain their relatively favorable clinical course. We noted that these misclassified tumors rarely exhibit SCNA features commonly associated with IDH-wildtype glioblastoma such as + 7/ − 10 and *EGFR* amplification. It remains an open question whether histological WHO grade 4 IDH-wildtype glioblastoma without molecular features of IDH-wildtype glioblastoma should be considered less aggressive than their counterparts, but our results support this notion [23].

## Conclusions

The primary contribution of this work is the development of a computational tool that accurately classifies the molecular subtype of patients’ tumors in retrospective adult diffuse glioma studies that have available SCNA data. We identified all patients with likely 1p/19q-codeleted oligodendrogliomas, IDH-wildtype glioblastomas, and IDH-mutant astrocytomas in the REMBRANDT study in an effort to make the REMBRANDT study a better resource for validating diffuse glioma research. We also propose evidence-based thresholds for calling 1p/19q-codeletions and + 7/ − 10 from gene-level SCNA data.

## Supplementary Information

**Additional file 1.** Additional tables.

**Additional file 2.** Additional figures.

## Data Availability

All code and trained models are made available for public use at github.com/nknuecht/scna2idh. All data is publicly available.
