# Supplementary material for: Machine learning modeling of genome-wide copy number alteration signatures reliably predicts IDH mutational status in adult diffuse glioma
Source: Acta Neuropathol Commun. 2021 Dec 4;9:191. doi: 10.1186/s40478-021-01295-3 (PMC8645099; doi:10.1186/s40478-021-01295-3)
Supplement: Supplementary file 2 — Additional file 2. Additional figures. [file 40478_2021_1295_MOESM2_ESM.pdf]

SUPPLEMENTARY FIGURES

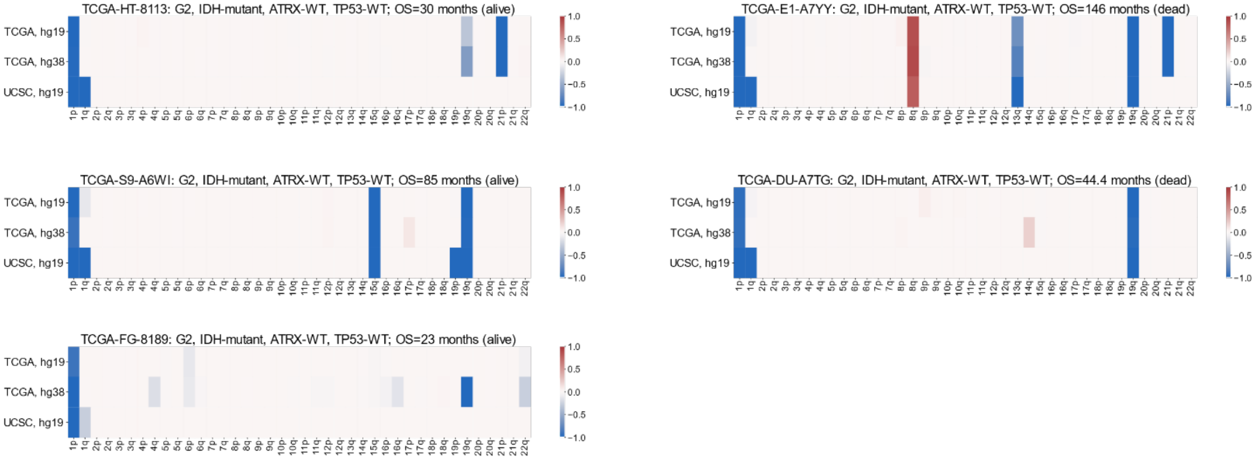

SUPPLEMENTAL FIGURE 1. SCNA Profiles of excluded TCGA IDH-mutant tumors. In four of the five TCGA IDH-mutant samples we excluded from our study, the UCSC hg19 pipeline called monosomy of chromosome 1 while the other pipelines called 1q intact. In one case, the GDC hg38 pipeline called loss of 19q, but the other two pipelines did not. None of these patients harbored *TP53* or *ATRX* mutations, common in IDH-mutant astrocytomas, and thus we suspect they are oligodendrogliomas despite their copy number profile not consistently support that claim.

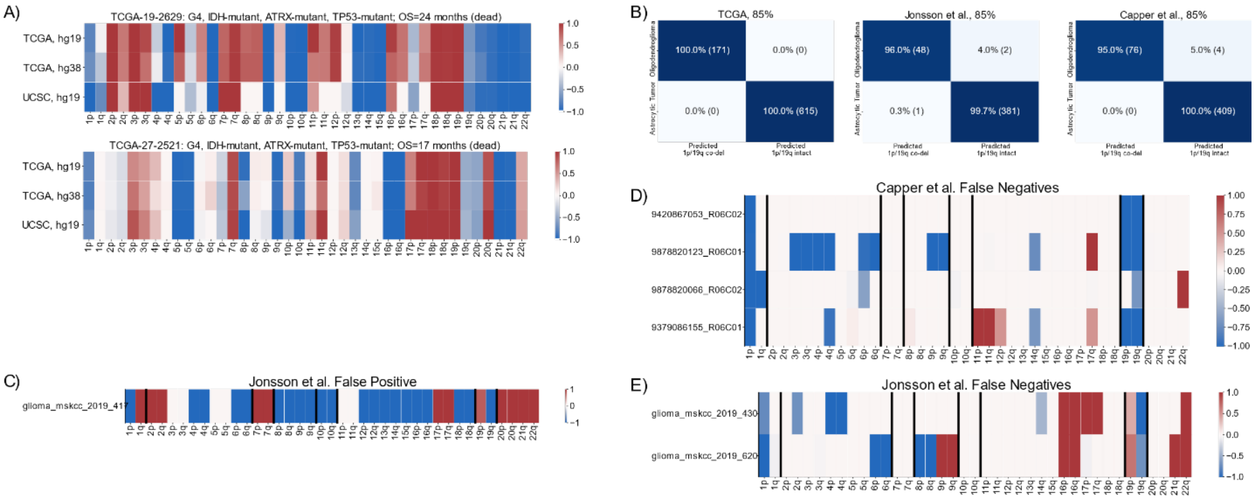

SUPPLEMENTAL FIGURE 2. Development and validation of 1p/19q-codeletion threshold. A) SCNA profiles of two TCGA grade 4 IDH-mutant astrocytomas that would have been considered 1p/19q-codeleted at thresholds lower than 85% (75% and 80%). **B)** Confusion matrixes show our 1p/19q-codeletion screen on the TCGA training set and validation sets published by Jonsson et al. and Capper et al. were accurate. **C)** Of the 921 astrocytic tumors in the validation sets published by Jonsson et al. and Capper et al., our 1p/19q co-deletion screen produced only 1 false positive. This patient is a grade 4 IDH-mutant astrocytoma that harbors high copy number alteration burden, including loss of 1p and 19q, co-gain of 1q and 19p, and +7/-10. We recommend examining the SCNA profiles of SCNA-burdened tumors predicted to be 1p/19q-codeleted. **D)** Four of the six false negative tumors from our 1p/19q-codeletion validation screen are from the dataset published by Capper et al. SCNA data indicates that each of these tumors harbored monosomy of chromosome 1 and/or monosomy of chromosome 19, and therefore are not 1p/19q-codeleted. Thus, these tumors do not represent model errors—they represent either misdiagnosed patients or SCNA pipeline errors. **E)** The remaining two false negative tumors from our 1p/19q-codeletion screen validation fail to meet our 85% threshold: one meets a threshold of 75% and the other meets a threshold of 71%.

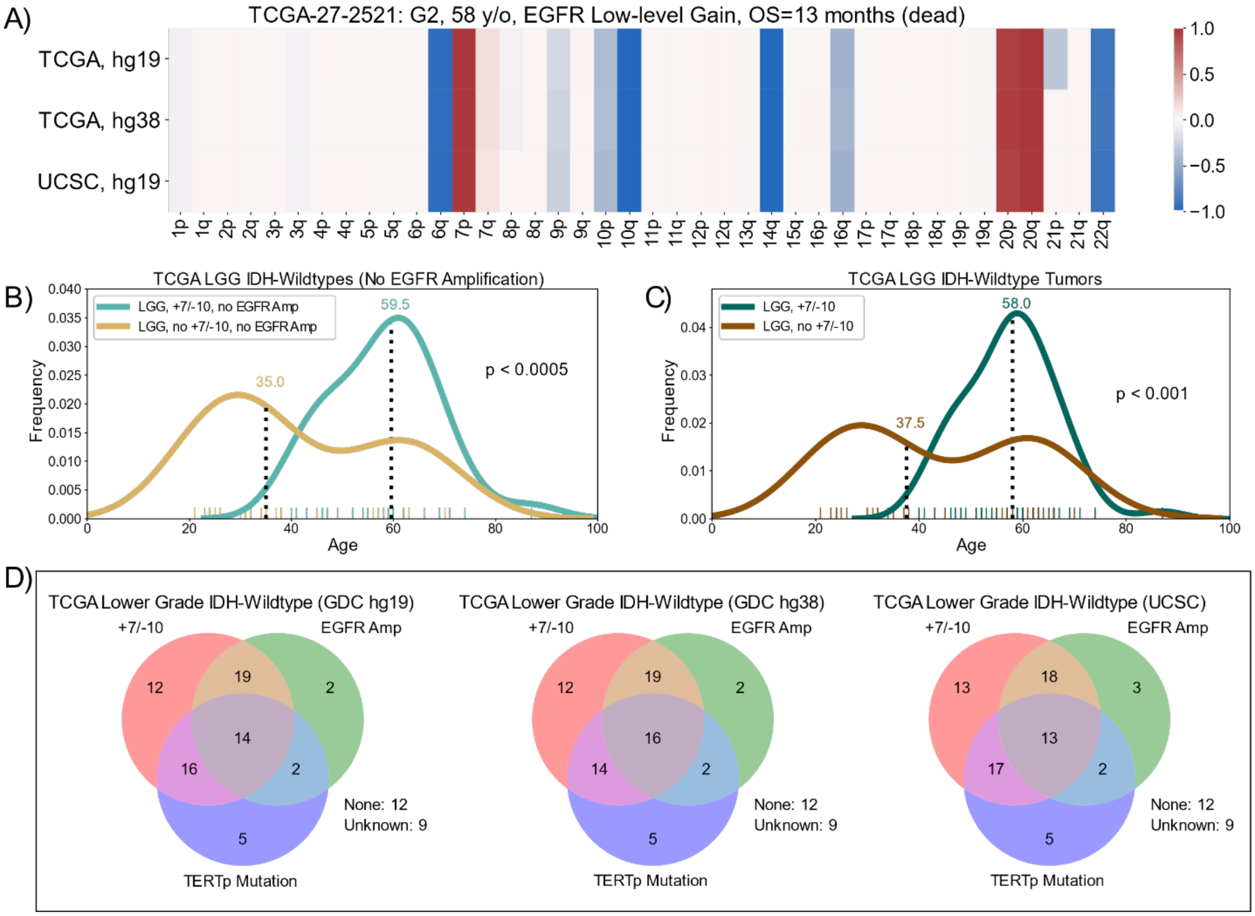

SUPPLEMENTAL FIGURE 3. SCNA profiles, age associations, and training set inclusion implications of our +7/-10 threshold. A) The SCNA profile of the TCGA histological lower-grade IDH-wildtype glioma without *EGFR* amplification closest to our 50% threshold for +7/-10. **B)** Among TCGA histological lower grade IDH-wildtype glioma without *EGFR* amplification, those with +7/-10 are significantly older than those without +7/-10. **C)** Among TCGA histological lower-grade IDH-wildtype glioma *with or without EGFR* amplification, those with +7/-10 are significantly older than those without +7/-10. **D)** The SCNA profile of the TCGA histological lower grade IDH-wildtype glioma with no *TERT*p mutation, no +7/-10, and with inconsistent *EGFR* amplification status across three SCNA pipelines. **E)** The classification of TCGA histological lower-grade IDH-wildtype glioma as pediatric-type or IDH-wildtype glioblastoma varies based on SCNA pipeline choice. The source of this variation is *EGFR* amplification calling inconsistency.

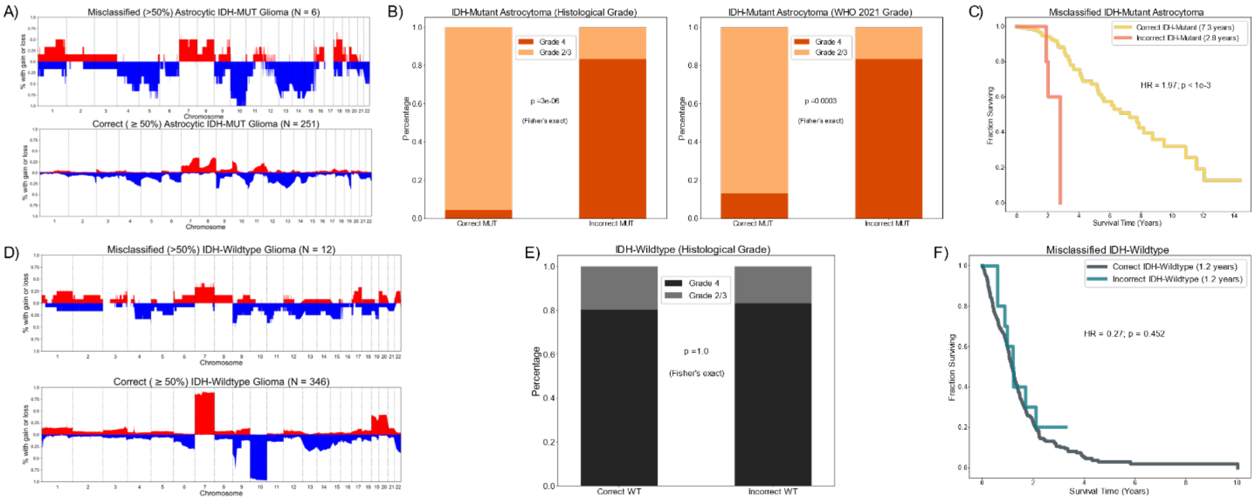

SUPPLEMENTAL FIGURE 4. Differences between correct and incorrect IDH mutation predictions on TCGA astrocytic tumors during cross validation. A) Misclassified IDH-mutant astrocytomas had increased copy number burden, especially on chromosome 10. **B)** Misclassified IDH-mutant astrocytomas were disproportionately grade 4 before and after lower grade IDH-mutant astrocytomas with *CDKN2A* homozygous deletion were promoted to grade 4 in accordance with the guidelines proposed for the fifth edition of the WHO classification of CNS tumors. **C)** Misclassified IDH-mutant astrocytomas followed a clinical course significantly worse than correctly classified IDH-mutant astrocytomas. **D)** Misclassified IDH-wildtype glioblastomas tended to have fewer SCNAs than their correctly classified counterparts, especially on chromosomes 7 and 10. **E)** No difference in histological grade between correctly and incorrectly classified IDH-wildtype glioblastomas was observed. **F)** No difference in patient outcome between correctly and incorrectly classified IDH-wildtype glioblastomas was observed.

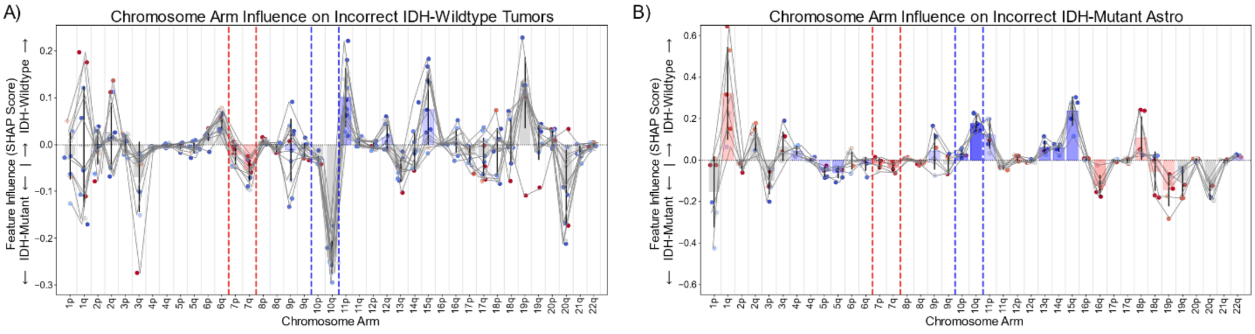

SUPPLEMENTAL FIGURE 5. Atypical chromosome arm 10q SCNA status drives IDH mutation classifier mistakes during cross validation. A) A SHAP interpretation of the IDH mutation classifier's mistakes on IDH-wildtype tumors indicates that intact 10q status along with low-level gains of on 7q drove incorrect predictions. Lines are drawn connecting points associated with the same patient. Each incorrectly predicted patient is assigned a point per chromosome arm. The magnitude of each point's vertical coordinate is an indication of how influential the chromosome arm was to the classifier's prediction. Positive vertical values favor IDH-wildtype predictions; negative values indicate a preference for IDH-mutant astrocytoma predictions. The color of each point corresponds to the average chromosome arm SCNA value: blue indicates loss, and red indicates gain. The magnitude of the bars drawn for each chromosome arm is the average chromosome arm SHAP score for all patients plotted; the bar's color indicates the average SCNA state across all patients plotted. **B)** A SHAP interpretation of the IDH mutation classifier's mistakes on IDH-mutant astrocytomas indicates that losses on 10q and 15q and gains on chromosome arm 1q drove incorrect predictions.

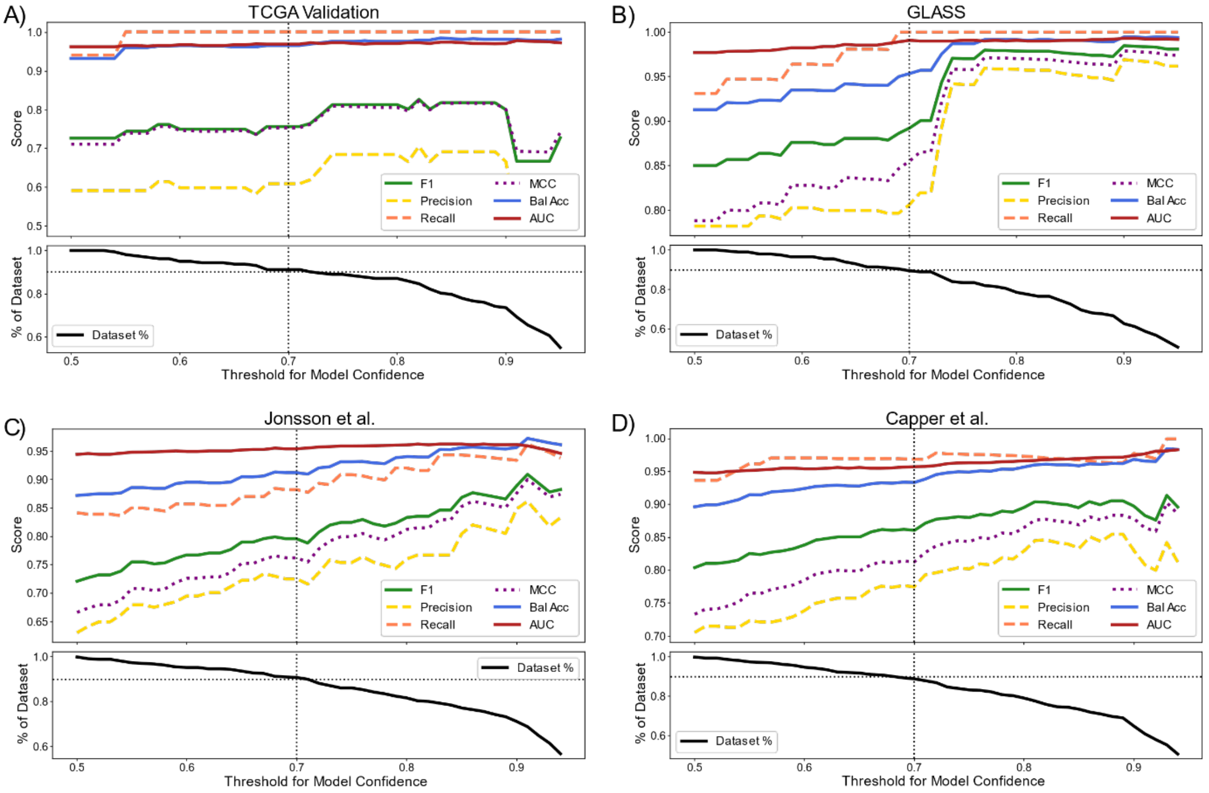

SUPPLEMENTAL FIGURE 6. Model calibration increases performance of the astrocytic IDH-mutation classifier across four validation sets. Model calibration shows that performance of our astrocytic IDH-mutation classifier increases with prediction confidence in four validation sets: the holdout TCGA validation set **(A)**, the GLASS dataset **(B)**, and datasets published Jonsson et al. **(C)** and Capper et al. **(D)**.

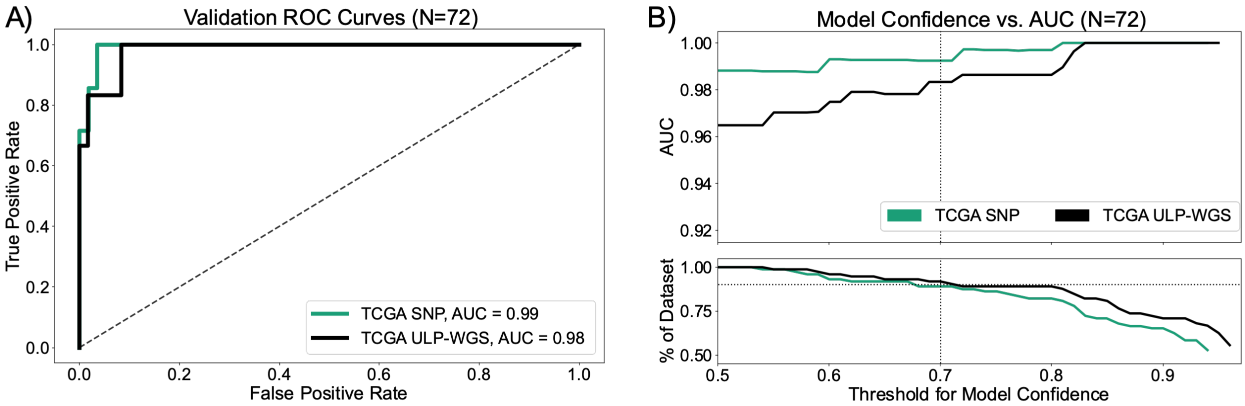

SUPPLEMENTAL FIGURE 7. Validation results for the IDH mutation classifier for TCGA SNP array derived and ultra-low-pass whole genome sequencing (ULP-WGS) derived SCNA data. A) IDH mutation classifier ROC curves for TCGA patients with computed ULP-WGS derived SCNA data. The high confidence predictions (confidence > 70%) based on SNP derived and ULP-WGS derived SCNA data were very similar: one astrocytic tumor with ULP-WGS-like data in the TCGA validation set was predicted differently (ULP-WGS AUC=0.98, SNP AUC=0.99). **B)** Furthermore, disregarding lower-confidence predictions (confidence < 70%) made from ULP-WGS derived SCNA data does not exclude more patients than from SNP derived SCNA data (10% for each).

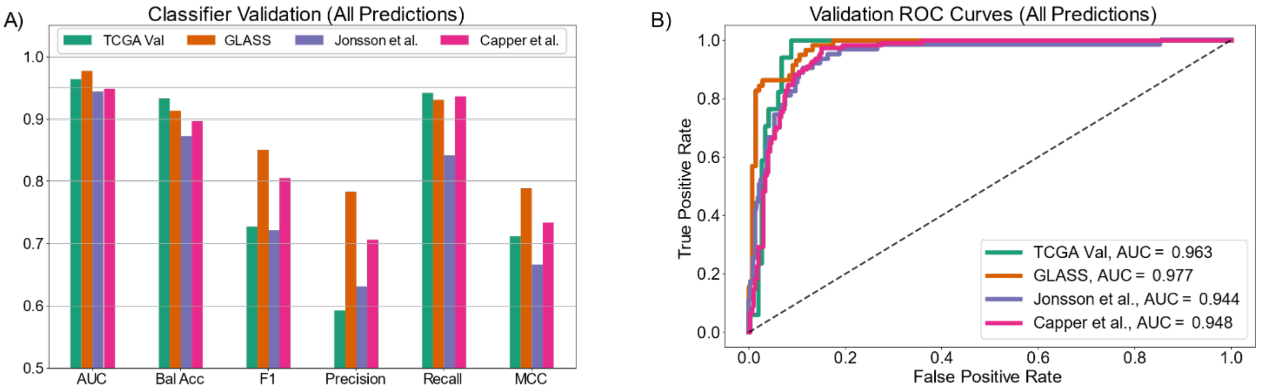

SUPPLEMENTAL FIGURE 8. Validation results for the IDH mutation classifier for all samples across four validation sets. A) IDH mutation classifier results for predictions over all patients and validation sets. The model performed well across all metrics except for precision, indicating that IDH-wildtype tumors were more difficult to identify than IDH-mutation astrocytomas. **B)** AUC scores were slightly lower than those achieved by evaluating the IDH-mutation classifier on predictions with 70% confidence or greater, but they were still above 0.94 on all four validation sets.

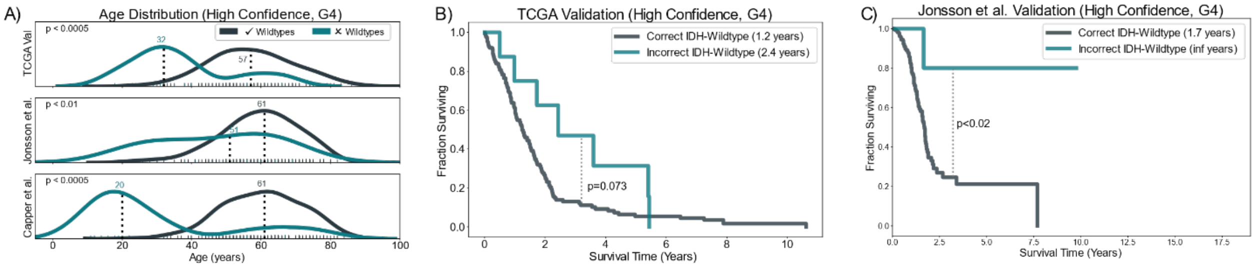

SUPPLEMENTAL FIGURE 9. Misclassified histological grade 4 IDH-wildtype tumors in our validation set were younger and survived longer than their correctly classified counterparts. A) When our age and survival analysis is restricted to histological grade 4 IDH-wildtype glioblastomas, tumors misclassified by our IDH-mutation classifier remained significantly younger than correctly classified tumors across three validation sets. **B)** Because our TCGA validation set is entirely composed of grade 4 tumors, misclassified IDH-wildtype tumors in this dataset survive better than correctly classified IDH-wildtype tumors with the same marginal significance reported in the main text. **C)** On the dataset published by Jonsson et al., correctly misclassified histological grade 4 IDH-wildtype tumors survived significantly better than correctly classified histological grade 4 IDH-wildtype tumors.

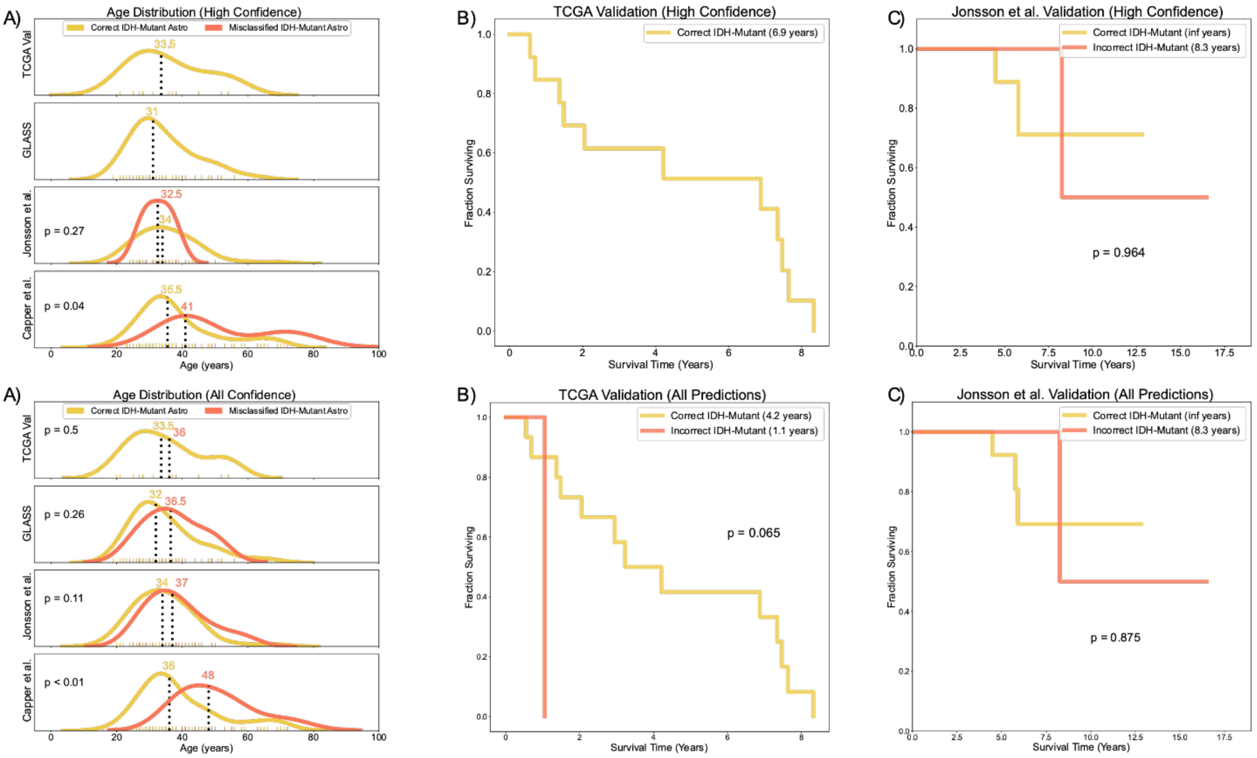

SUPPLEMENTAL FIGURE 10. No consistent age differences between correctly and incorrectly classified IDH-mutant astrocytomas were observed. A) Among high confidence predictions, we did not observe a consistent, significant age difference between correctly and incorrectly predicted IDH-mutant astrocytomas over three independent validation sets and the holdout TCGA validation set. **B, C)** Among high confidence validation predictions in our TCGA holdout validation set and a dataset published by Jonsson et al., we did not observe a significant difference in patient outcome between correctly and incorrectly predicted IDH-mutant astrocytomas. **D)** Over all patient predictions, we did not observe a consistent, significant age difference between correctly and incorrectly predicted IDH-mutant astrocytomas over three independent validation sets and the holdout TCGA validation set. **E, F)** Over all patient predictions in our TCGA holdout validation set and a dataset published by Jonsson et al., we did not observe a significant difference in patient outcome between correctly and incorrectly predicted IDH-mutant astrocytomas.

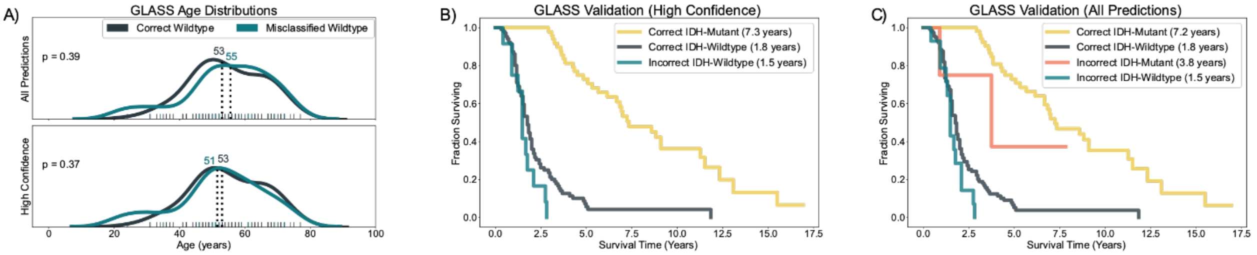

SUPPLEMENTAL FIGURE 11. Patients in the GLASS dataset do not follow the same age or survival patterns found in the other validation datasets. A) No significant age difference between correct and incorrectly classified IDH-wildtype glioblastomas was observed in the GLASS dataset. **B, C)** The tendency for misclassified IDH-wildtype glioblastomas to outlive correctly classified IDH-wildtype glioblastomas was not observed in the GLASS dataset.

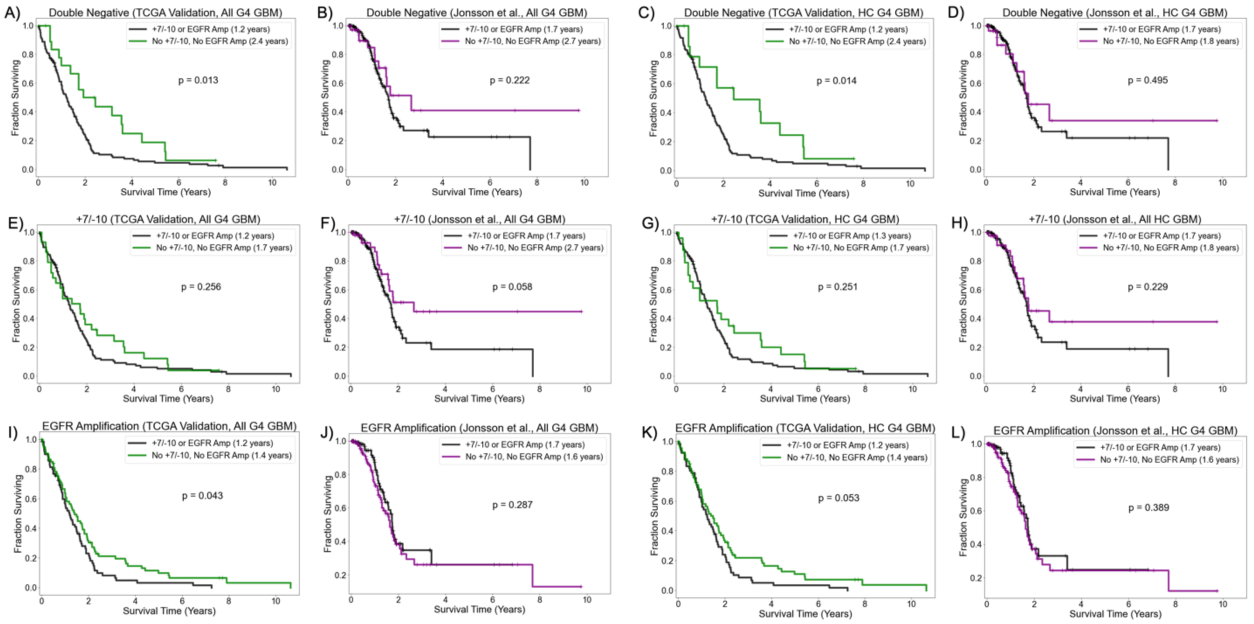

SUPPLEMENTAL FIGURE 12. Survival differences incurred by +7/-10 and EGFR amplification in the TCGA validation set and the Jonsson et al. validation set are inconsistent among histological grade 4 IDH-wildtype glioblastoma both when examining all such tumors (All) and when examining only those predicted by our model with high confidence (HC). A-B) Among all available histological grade 4 IDH-wildtype glioblastomas in the TCGA validation and the Jonsson et al. dataset, double negative patient (no +7/-10, no EGFR amplification) do significantly better in the TCGA validation dataset than patients with either +7/-10 or EGFR amplification; this is not true for similar patients in the Jonsson et al. validation dataset. **C-D)** Among histological grade 4 IDH-wildtype glioblastomas in the TCGA validation and the Jonsson et al. dataset that our model predicted with high confidence (HC) (confidence > 70%), double negative patient (no +7/-10, no EGFR amplification) do significantly better in the TCGA validation dataset than patients with either +7/-10 or EGFR amplification; this is not true for similar patients in the Jonsson et al. validation dataset. **E-F)** Among all available histological grade 4 IDH-wildtype glioblastomas in the TCGA validation and the Jonsson et al. dataset, patients lacking +7/-10 do not do significantly better in the TCGA validation dataset than patients with +7/-10; in the Jonsson et al. dataset, patients lacking +7/-10 have marginally significantly better outcomes. **G-H)** Among histological grade 4 IDH-wildtype glioblastomas in the TCGA validation and the Jonsson et al. dataset that our model predicted with HC, patients lacking +7/-10 do not do significantly better in the TCGA validation dataset or Jonsson et al. validation dataset. **I-J)** Among all available histological grade 4 IDH-wildtype glioblastomas in the TCGA validation and the Jonsson et al. dataset, patients lacking EGFR amplification do better in the TCGA validation dataset but not in the Jonsson et al. dataset. **K-L)** Among histological grade 4 IDH-wildtype glioblastomas in the TCGA validation and the Jonsson et al. dataset that our model predicted with HC, patients lacking EGFR amplification do not do marginally significantly better in the TCGA validation dataset but not in the Jonsson et al. validation dataset.

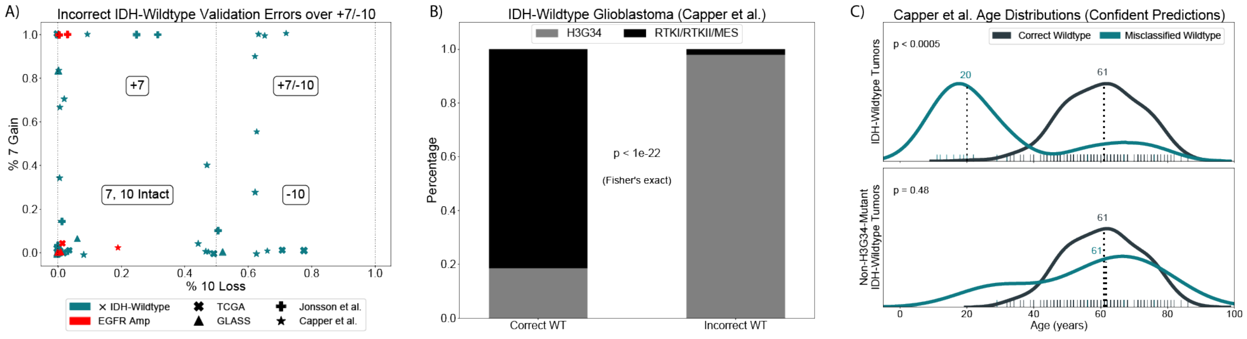

SUPPLEMENTAL FIGURE 13. Distribution of IDH-wildtype glioblastoma SCNA features and H3 G34-mutant gliomas in misclassified IDH-wildtype tumors. A) Of all misclassified histological WHO grade 4 IDH-wildtype gliomas in our three independent validation sets and the holdout TCGA validation set, only 19% (11/59) show molecular IDH-wildtype glioblastoma features +7/-10 or EGFR amplification. Point size indicates prediction confidence. **B)** In the dataset published by Capper et al., 88% of misclassified IDH-wildtype samples were WHO grade 4 diffuse hemispheric glioma, H3 G34-mutant, while only 2% of correctly classified samples were diffuse hemispheric glioma, H3 G34-mutant. **C)** The presence of diffuse hemispheric glioma, H3 G34-mutant in the dataset published by Capper et al. explained the age difference between correctly and incorrectly classified IDH-wildtype glioblastomas.

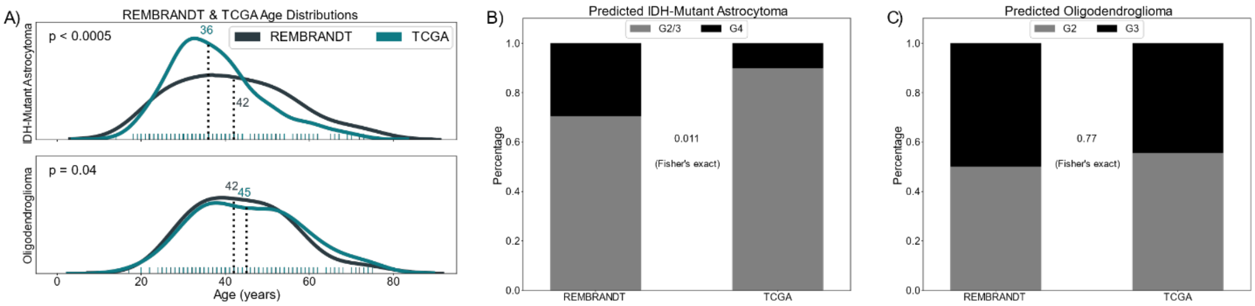

SUPPLEMENTAL FIGURE 14. Predicted REMBRANDT IDH-mutant astrocytomas harbor more risk factors than their TCGA counterparts, but the same is not true for predicted 1p/19q-codeleted oligodendroglioma. A) Predicted IDH-mutant astrocytomas in the REMBRANDT dataset are significantly older than ground truth IDH-mutant astrocytomas in our TCGA training set. Predicted 1p/19q-codeleted oligodendrogliomas are younger than ground truth 1p/19q-codeleted oligodendrogliomas in our TCGA training set, but the difference in median age is only 3 years. **B)** Predicted IDH-mutant astrocytomas in the REMBRANDT dataset are disproportionally grade 4 compared to ground truth IDH-mutant astrocytomas in our TCGA training set. **C)** Predicted 1p/19q-codeleted oligodendrogliomas and ground truth 1p/19q-codeleted oligodendrogliomas in our TCGA training set have a similar proportion of grade 2 and grade 3 tumors.

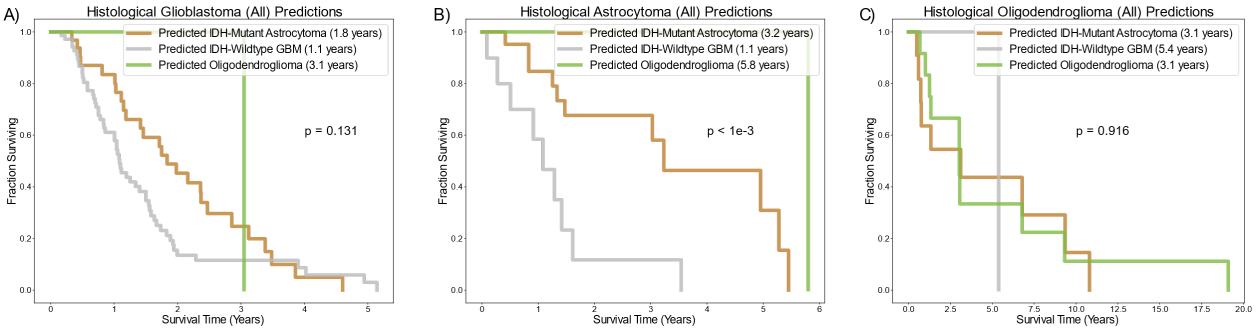

SUPPLEMENTAL FIGURE 15. Survival results for model predictions on all REMBRANDT patients. Kaplan–Meier curves are given for predicted adult diffuse glioma molecular subtype diagnosis of REMBRANDT patients with histological diagnosis of glioblastoma **(A)**, astrocytoma **(B)**, and oligodendroglioma **(C).**

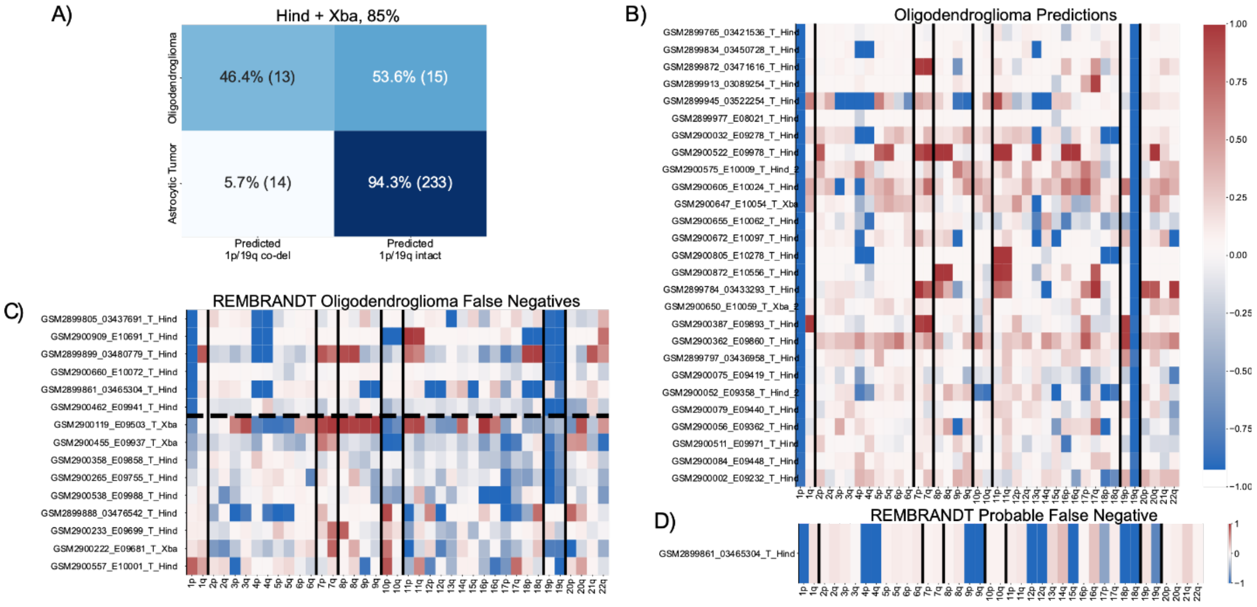

SUPPLEMENTAL FIGURE 16. Our 1p/19q-codeletion predictions were accurate on the REMBRANDT dataset despite discordance with histological diagnosis. A) Only 46% of REMBRANDT histological oligodendrogliomas were predicted to harbor 1p/19q co-deletions, and only 48% of REMBRADNT tumors harboring predicted 1p/19q co-deletions (n=27) were diagnosed as histological oligodendroglioma (n=13). **B)** Copy number profiles of predicted oligodendroglioma revealed clear 1p/19q co-deletions without loss of 1q or 19p. **C)** Of patients diagnosed with histological oligodendroglioma for whom we did not predict 1p/19q co-deletions, we saw that most (5/6, above dotted line) tumors that lose 1p harbor monosomy chromosome 19, prohibiting a 1p/19q-codeletion. **D)** SCNA profile of a likely misclassified 1p/19q-codeleted oligodendroglioma. This patient’s proportion of 19q loss falls slightly below our 85% threshold (79%).

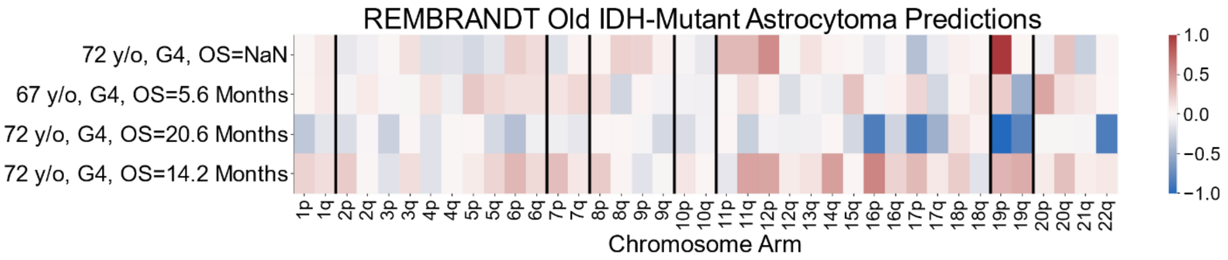

SUPPLEMENTAL FIGURE 17. The oldest predicted REMBRANDT IDH-mutant astrocytomas do not have IDH-wildtype glioblastoma-like SCNA profiles. Pictured here are SCNA profiles of four older (age range 65-80) patients diagnosed with predicted IDH-mutant astrocytoma. None harbor +7/-10 and only one shows EGFR amplification (72 y/o, G4, OS=20.6 Months).
